# The multifaceted impact of physical exercise on FoxO signaling pathways

**DOI:** 10.3389/fcell.2025.1614732

**Published:** 2025-08-08

**Authors:** Wei Liu, Pengjun Meng, Zhi Li, Yifei Shen, Xin Meng, Shen Jin, Mengdi Hu

**Affiliations:** ^1^ Department of Physical Education, Xidian University, Xi’an, Shaanxi, China; ^2^ Office of Scientific Research, Ersha Sports Training Center of Guangdong Province, Guangzhou, Guangdong, China; ^3^ Gynecology Department, Shaanxi Provincial Hospital of Chinese Medicine, Xi’an, Shaanxi, China

**Keywords:** FoxO signaling pathways, physical exercise, metabolic regulation, skeletal muscle, complex, left

## Abstract

This review explores the multifaceted impact of physical exercise on FoxO signaling pathways, which play a central role in cellular homeostasis, stress response, metabolism, and longevity. Exercise influences FoxO proteins—particularly FoxO1, FoxO3, FoxO4, and FoxO6—through diverse mechanisms, including phosphorylation, acetylation, and ubiquitination, determining their localization, transcriptional activity, and stability. Regular exercise modulates FoxO signaling by activating pathways like PI3K/AKT, AMPK, SIRT1, and IGF-1, promoting cellular resilience against oxidative stress, apoptosis, and metabolic dysfunction. The review highlights how exercise-induced modulation of FoxO pathways contributes to improved insulin sensitivity, muscle hypertrophy, cardiovascular health, neuroprotection, and reduced risks of chronic diseases, including metabolic syndrome, neurodegeneration, cardiovascular disease, and cancer. Additionally, it addresses the role of exercise in preventing muscle atrophy under various conditions, such as pharmacological interventions, aging, disease, and dietary factors. By enhancing FoxO signaling, exercise promotes anabolic processes, mitochondrial function, autophagy, and antioxidant defenses. Understanding the intricate relationship between exercise and FoxO pathways offers insights into developing therapeutic strategies to mitigate disease progression.

## Graphical Abstract

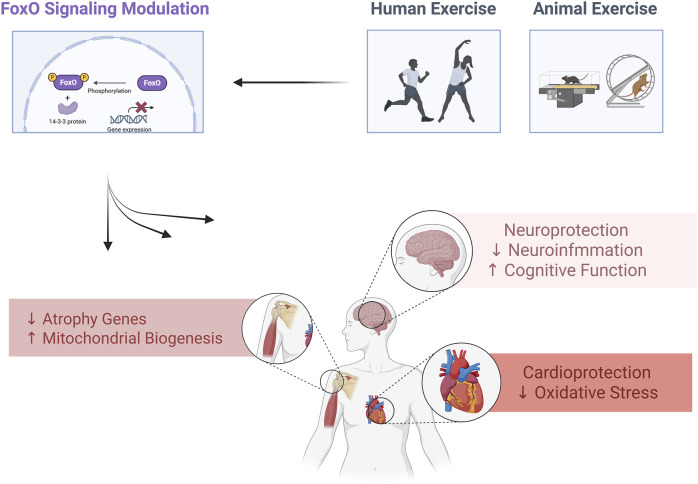

## Highlight

• Physical exercise modulates FoxO pathways regulating health and disease processes.
• Exercise enhances FoxO activity, improving metabolism, stress resistance, and longevity.
• FoxO modulation by exercise prevents muscle atrophy, neurodegeneration, and inflammation.
• Exercise-induced FoxO signaling boosts insulin sensitivity and cardiovascular health.
• FoxO regulation through exercise supports antioxidant defenses and mitochondrial function.

## 1 Introduction

The Forkhead box O (FoxO) family of transcription factors integrates diverse signaling pathways to regulate cellular homeostasis, stress responses, and longevity. The PI3K/AKT pathway is central to their regulation, which phosphorylates FoxO proteins, promoting cytoplasmic retention and inhibiting their transcriptional activity. Conversely, dephosphorylated FoxO translocates to the nucleus, activating genes involved in apoptosis, cell-cycle arrest, and oxidative stress resistance. Beyond PI3K/AKT, FoxO activity is modulated by Ras-MEK-ERK signaling, growth factors, and cytokines, highlighting their role as signaling integrators (Zhang and Zhang, 2019; Farhan et al., 2017).

FoxO proteins also interact with pathways like insulin/IGF-1, where serotonin modulates stress responses (Liang et al., 2006), and SIRT1/FoxO signaling in diabetic nephropathy, linking metabolism to cell proliferation and fibrosis (Zhong and Zhang, 2024). Emerging roles in tumorigenesis reveal dual functions of FoxO as a tumor suppressor or oncogene, depending on context. Therapeutically, targeting FoxO pathways offers promise in cancer and metabolic disorders (Arden, 2004). These dynamic regulators thus bridge cellular signaling, disease pathogenesis, and potential therapeutic strategies.

Physical exercise modulates cellular signaling pathways across multiple organ systems, enhancing health and functional capacity. In metabolic regulation, exercise activates the PI3K/AKT pathway via insulin/IGF-1 signaling, promoting GLUT4 translocation to increase glucose uptake in muscles (Pinches et al., 2022). This improves insulin sensitivity and metabolic homeostasis. In cardiovascular health, aerobic exercise stimulates neuregulin1 signaling, enhancing cardiac contractility and endothelial nitric oxide (NO) production through eNOS activation, which supports vasodilation and vascular repair (Chen et al., 2022).

Exercise also drives skeletal muscle adaptations by altering signaling networks that regulate hypertrophy, metabolic shifts, and tissue remodeling. Concurrently, it enhances neuronal resilience by upregulating neurotrophic factors like BDNF, fostering synaptic plasticity and neurogenesis. At the molecular level, exercise induces systemic changes in RNA, proteins, and metabolites, promoting DNA repair mechanisms in tissues such as the colon (Fowler et al., 2024; Consorti et al., 2021). By modulating these pathways, exercise mitigates age-related decline, reduces chronic disease risks, and extends healthspan. These effects underscore its role as a non-pharmacological intervention to optimize cellular function and combat pathologies linked to metabolic, cardiovascular, and neurological disorders.

This narrative review examines the complex interplay between physical exercise and FoxO signaling pathways, highlighting their critical roles in cellular homeostasis, aging, disease prevention, and therapeutic potential. It emphasizes the molecular mechanisms of FoxO regulation and describes how different exercise modalities influence FoxO activity in skeletal muscle, cardiac tissue, and the nervous system. The review further addresses exercise-induced FoxO modulation in metabolic syndrome, insulin sensitivity, neurodegeneration, cardiovascular diseases, inflammation, and cancer, underlining the broad systemic impact of this signaling pathway.

## 2 FoxO signaling: molecular mechanisms and physiological functions

**FIGURE 1:**
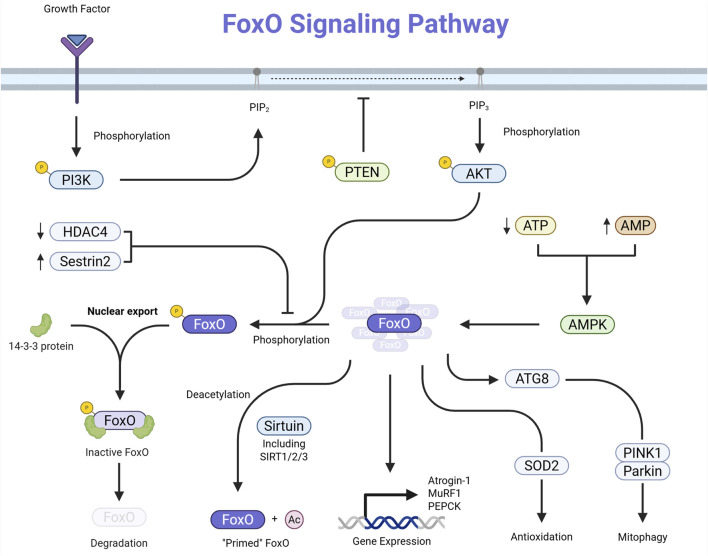
Schematic representation of the FoxO signaling pathway. Growth-factor or insulin binding activates PI3K, which converts PIP_2_ to PIP_3_. PIP_3_ recruits and activates AKT, which phosphorylates FoxO transcription factors in the nucleus. Phosphorylated FoxO proteins bind 14-3-3 and are exported to the cytoplasm, inhibiting their transcriptional activity. Phosphorylated FoxO may also undergo proteasomal degradation. PTEN opposes PI3K activity by converting PIP_3_ back to PIP_2_, thus providing negative regulation of this pathway.

The FoxO family of proteins comprises transcription factors—namely FoxO1, FoxO3, FoxO4, and FoxO6—integral to regulating various cellular processes such as apoptosis, cell cycle control, glucose metabolism, oxidative stress resistance, and longevity. FoxO proteins are subject to regulation through multiple post-translational modifications, including phosphorylation, acetylation, and ubiquitination. These modifications influence their subcellular localization, DNA-binding affinity, and stability, thereby modulating their transcriptional activity (Lees et al., 2023; Beretta et al., 2019) (Figure 1).

Growth factors and insulin activate pathways like PI3K/AKT, leading to the phosphorylation of FoxO proteins. This phosphorylation induces a conformational change that facilitates binding to 14-3-3 proteins, resulting in the export of FoxO factors from the nucleus to the cytoplasm and a subsequent reduction in their transcriptional activity (Manning and Toker, 2017; Vogt et al., 2005). Also, acetylation of FoxO proteins can alter their DNA-binding ability and sensitivity to phosphorylation. Enzymes like SIRT1 can deacetylate FoxO factors, influencing their activity and stability (Vogt et al., 2005; Wang et al., 2012).

Additionally, ubiquitination targets FoxO proteins for proteasomal degradation, thus controlling their levels within the cell (Vogt et al., 2005). Active FoxO transcription factors regulate the expression of genes involved in several critical cellular functions. They can induce the expression of pro-apoptotic genes, thereby promoting programmed cell death in response to cellular stress or damage (Yadav et al., 2018).

They also upregulate cyclin-dependent kinase inhibitors, leading to cell cycle arrest and allowing for DNA repair or stress adaptation (Lees et al., 2023). In hepatic cells, FoxO factors increase the expression of enzymes such as phosphoenolpyruvate carboxykinase and glucose-6-phosphatase, which are involved in gluconeogenesis and glycogenolysis, thereby influencing glucose metabolism (Salih and Brunet, 2008).

Additionally, FoxO proteins enhance the expression of antioxidant enzymes, contributing to cellular defense against oxidative damage (Dumitrascu and Bucur, 2013). In model organisms like *Caenorhabditis elegans*, the FoxO homolog DAF-16 has been shown to activate genetic pathways associated with increased lifespan (Sun et al., 2017). FoxO factors interact with various signaling pathways to coordinate cellular responses. FoxO and Notch pathways regulate muscle differentiation, with FoxO1 promoting corepressor exchange at specific gene promoters (Kitamura et al., 2007).

Also, energy-sensing AMPK directly phosphorylates FoxO transcription factors at multiple regulatory sites. This phosphorylation enhances transcriptional activity of FoxO, leading to the expression of genes involved in stress resistance and metabolic adaptation (Greer et al., 2009).

## 3 Impact of exercise on FoxO pathways in skeletal muscle

We have summarized the study findings regarding effects of exercise on FoxO pathway in skeletal muscles in Supplementary Table S1.

### 3.1 Pharmacologically‐induced muscle atrophy

Across glucocorticoid, chemotherapeutic, and metabolic-drug models, exercise invariably protects skeletal muscle by re-establishing FoxO3a Ser^253^ phosphorylation, driving the transcription factor out of the nucleus and relieving the MuRF1/Atrogin-1 axis. What distinguishes one model from another is which upstream brake is recruited and whether parallel catabolic routes (autophagy, MSTN/Smad) are also restrained (Supplementary Table S1).

In rats treated with dexamethasone, running and resistance lifting decrease HDAC4-mediated deacetylation of FoxO3a, restoring its phosphorylation and suppressing MuRF1/Atrogin-1 expression (Liang et al., 2025). Resistance work goes a step further and upregulates Sestrin2, an AMPK–AKT facilitator that blocks FoxO3a nuclear import even when HDAC4 activity persists (Yang Y. et al., 2023). These observations imply that exercise modality selects distinct regulators (HDAC4 versus Sestrin2) while converging on the same FoxO3a endpoint.

In mice injected with cisplatin, treadmill or weighted-ladder protocols reinstate AKT/PGC-1α signaling, rebound FoxO3a phosphorylation, and limit MuRF1/Atrogin-1 induction (Bae et al., 2021; Sakai et al., 2017). Yet only one study reports concordant repression of autophagy genes such as Beclin-1, BNIP3, and LC3-II (Bae et al., 2021), highlighting a methodological controversy. Autophagy control appears sensitive to drug dose, exercise intensity, and sampling time, whereas FoxO inhibition is comparatively robust.

In rats subjected to chronic doxorubicin exposure, endurance exercise normalizes FoxO3a phosphorylation but leaves MuRF1 or autophagy markers unchanged, suggesting a FoxO-independent proteolytic drive (Kwon, 2020). By contrast, in an acute doxorubicin toxicity model, short-term running cuts FoxO1/FoxO3 mRNA and BNIP3 expression, a response attributed to a surge in PGC-1α, an established FoxO corepressor (Kavazis et al., 2014). Thus, time-course (acute vs. chronic) rather than exercise *per se* dictates whether downstream atrogenes track with FoxO modulation.

In mice receiving dapagliflozin, resistance training lowers the total abundance of FoxO1/3a proteins, not merely their phosphorylation, and rescues mTORC1-anabolic signaling. It preserves muscle despite persistent glycosuria (Yang X. et al., 2023). This finding broadens the catalogue of exercise-controlled nodes from post-translational phosphorylation to outright protein turnover.

**FIGURE 2:**
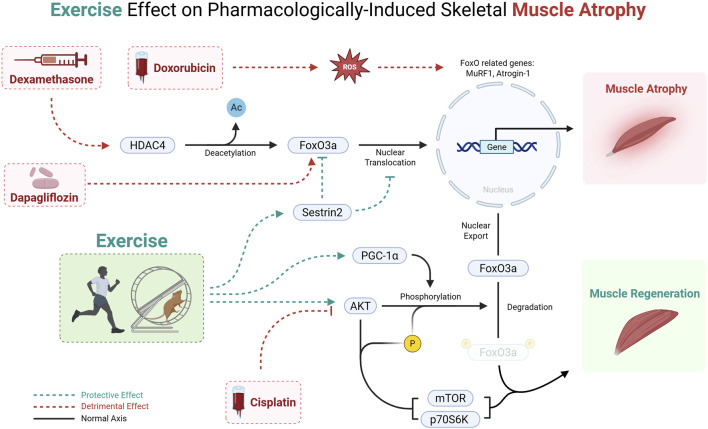
Mechanism of pharmacologically induced muscle wasting and the protective effect of exercise via the FoxO3a pathway. Cisplatin, Doxorubicin, Dexamethasone, and Dapagliflozin promote muscle atrophy, whereas exercise training counters this process by enhancing FoxO phosphorylation and thereby preventing FoxO3a nuclear translocation. This leads to a suppression of FoxO‐dependent transcription of muscle atrophy‐related genes, ultimately mitigating muscle loss.

In summary (Figure 2), pharmacologically driven muscle‐wasting states share a common vulnerability. They all hinge on unchecked FoxO3a activity to amplify ubiquitin-proteasome and, variably, autophagy catabolism. Exercise consistently exploits this vulnerability by restoring FoxO3a phosphorylation and nuclear exclusion. It does so through drug- and modality-specific routes (HDAC4, Sestrin2, PGC-1α/TFEB, or accelerated FoxO protein turnover). These nuances matter clinically. Selecting the proper exercise method could target the dominant upstream inhibition for a given therapy. Thereby, it maximizes muscle preservation without pharmacologic add-ons. Nevertheless, the field is still performed in young-rodent models, and autophagy read-outs remain inconsistent, warnings that FoxO3a may not always be the final arbiter of drug-induced atrophy, especially in chronic or multimorbid settings. Bridging these gaps will require integrative studies in aged or diseased humans, systematic tracking of parallel catabolic circuits, and trials that match exercise dose and modality to the specific pharmacologic insult. Only then can we translate the elegant convergence on FoxO3a into reliable, personalized countermeasures against therapy-related muscle loss.

### 3.2 Disease, diet, and environment-related muscle atrophy

#### 3.2.1 Condition-related muscle atrophy

Exercise consistently modifies FoxO-centered networks across diverse catabolic settings. Yet, the direction and functional outcome of that modification are determined by the exercise modality (aerobic versus resistance), the disease milieu, and the species studied (Supplementary Table S1).

In diabetic mice, a modest treadmill loads preserved myofiber size without altering FoxO3a phosphorylation. This suggests that parallel anti-inflammatory signaling can bypass the canonical AKT/FoxO inhibition on proteolysis in a chronically hyperglycemic environment. NF-κB repression curtailed MuRF1 transcription, while a SIRT1/AMPK/PGC-1α axis boosted mitochondrial biogenesis and oxidative capacity, decreasing diabetes-induced wasting (Liu and Chang, 2018).

The same aerobic strategy is less effective when renal insufficiency superimposes metabolic acidosis and uremic toxins. In CKD mice, treadmill running mainly suppressed degradation pathways but did not fully reactivate protein synthesis (Wang et al., 2009). These data imply that endurance exercise protects muscle mainly by dampening inflammatory E3-ligase programs, with FoxO status becoming permissive rather than determinant.

Mechanical overload in CKD muscle and ladder climbing under hypoxia converge on FoxO phosphorylation and deacetylation, respectively. These two distinct biochemical locks keep the transcription factor out of the nucleus (Wang et al., 2009; Fu et al., 2024). In both cases, AKT (phosphorylation route) or SIRT2 (deacetylation route) neutralize FoxO-driven autophagy/atrophy genes, while mTOR/p70S6K or ribosomal biogenesis reactions reignite synthesis. Compared with endurance work, resistance loads act as a more proximal FoxO switch, directly tipping the balance towards hypertrophy even in a hostile metabolic or low-oxygen setting.

Not all elevations of FoxO show catabolism. Chronic wheel running in aged mice and swimming in *Caenorhabditis elegans* enhance AdipoR1/AMPK signalling that increases FoxO3a transcriptional activity yet simultaneously upregulates mitophagy, stem-cell renewal, and lifespan (Chen YL. et al., 2023). Here, FoxO serves as an organizer of quality-control autophagy rather than an executioner of proteolysis, illustrating a physiological “good FoxO” mode largely absent from the pathological rodent models above.

The sole clinical dataset in this context, ICU patients exposed to functional-electrical-stimulation (FES) assisted exercise, documents an early, global downregulation of FoxO target genes that FES fails to reverse (Fu et al., 2024). Persisting intramuscular inflammation and altered substrate use suggest that single-muscle activation is inadequate when systemic cytokinaemia, immobility, and impaired nutrient delivery coexist. Whether whole-body or combined aerobic-resistance regimens could restore a more anabolic FoxO signature in humans remains untested. Likewise, no trial has yet stratified patients by FoxO single-nucleotide variants or circulating adiponectin, which could dictate the responsiveness observed in rodents. Addressing these translational unknowns will require integrative protocols that pair exercise with anti-inflammatory or metabolic adjuncts and include mechanistic biopsies rather than relying solely on functional outcomes.

Taken together, the studies reveal three ways exercise intersects with FoxO signalling. (i) indirect suppression via upstream anti-inflammatory or metabolic cues (endurance in diabetes), (ii) direct post-translational blockade that re-routes substrates toward synthesis (resistance in CKD or hypoxia), and (iii) constructive activation that expands the autophagy–mitophagy toolkit for healthy ageing (lifelong spontaneous activity). The pre-clinical bias (five rodent studies versus one clinical) limits immediate extrapolation. Still, the diversity of responses already highlights why the “FoxO inhibition” paradigm is unlikely to succeed.

In summary, condition-related muscle atrophy is governed not by a uniform FoxO axis but by context-specific crosstalk between inflammatory, metabolic, and contractile cues. Aerobic exercise tends to spare muscle through anti-inflammatory routes that leave FoxO functionally inactive. Resistance loading neutralizes FoxO more directly and reinstates anabolism. Lifelong moderate activity can even harness FoxO-driven autophagy for healthspan extension. Limited human data underscore the need for trials integrating modality, intensity, and patient-specific biology, including FoxO genetics and circulating modulators, to convert these mechanistic insights into clinically actionable exercise prescriptions.

#### 3.2.2 Diet and lifestyle factors

Diet- and activity-based interventions converge on the FoxO family, yet the direction and consequences of that regulation differ sharply with exercise mode, species, and nutritional milieu (Supplementary Table S1).

Resistance training typically activates FoxO (nuclear, dephosphorylated) and turns it into a transcriptional hub for mitochondrial biogenesis and antioxidant defense. In climbing *Drosophila*, this activation drives PGC-1α, succinate dehydrogenase, and superoxide dismutase, preserving red-fiber integrity under salt stress and with ageing (Wen et al., 2023). A related Sirt1/FoxO axis in high-fat-fed flies lowers lipid peroxidation while restoring catalase activity (Jin et al., 2023). In aged rats, ladder-climbing or mixed resistance/aerobic work similarly reduces FoxO3a phosphorylation, permits nuclear entry, enhances autophagic flux, and lowers caspase-3-positive fibers (Zeng et al., 2020). Mechanical tension favors FoxO engagement in recycling damaged organelles and maintaining redox balance.

Conversely, most endurance exercises trigger phosphorylation-mediated FoxO inhibition. In young female rats, treadmill running plus extra-virgin olive oil elevates FoxO3a phosphorylation yet still upregulates Beclin-1, LC3, and Bnip3, implying that autophagy can be reinstated through alternate routes such as PGC-1α even when canonical FoxO transcription is dampened (Yeo et al., 2022). In zebrafish, swimming represses miR-128, activates IGF-1/PI3K/Akt, shifts FoxO3a to the cytoplasm, and restores mitochondrial respiration after D-galactose loading (Chen ZL. et al., 2023). Here, metabolic contexts that raise insulin or IGF-1 lean toward FoxO silencing, thereby restraining proteolysis while relying on other transcription factors (NRF-1, ERRα) for mitochondrial renewal.

Species differences are not trivial. Invertebrates show tight FoxO/Sirt1 coupling, whereas rodents rely more on AKT. *Drosophila* muscle benefits from chronic FoxO activity, but comparable activation in mammals can increase atrophy if AKT is low (e.g., fasting). Fiber-type composition also matters. Slow (type I) fibers tolerate FoxO activation better than glycolytic fibers because of higher basal autophagy and antioxidant buffering.

Overall, FoxO behaves less like an on/off switch and is regulated by AKT, AMPK, Sirt1, and upstream hormonal cues. Resistance exercise and caloric overload usually tilt the regulation toward activation to boost quality-control pathways. In contrast, endurance exercise, insulin elevation, or amino-acid surfeit tilt it toward suppression to spare protein and support metabolic flexibility. The apparent contradictions disappear once the upstream context is made explicit.

In summary, diet and lifestyle interventions regulate FoxO signalling along divergent pathways that depend on exercise modality, energetic state, and species-specific wiring. Resistance paradigms, especially under lipotoxic or osmotic stress, rely on FoxO activation to safeguard red-fiber quality. In contrast, endurance work often benefits from transient FoxO silencing coupled to alternative mitochondrial cues. Addressing the current shortage of well-controlled human studies remains essential for translating FoxO biology into therapies for diet- and age-related muscle decline.

### 3.3 Molecular and cellular mechanisms in muscle adaptation

#### 3.3.1 Transcriptomic and gene expression adaptations

Recent studies underscore the importance of FoxO proteins in mediating the balance between muscle protein synthesis and degradation under different exercise conditions (Supplementary Table S1).

In male mice, moderate-intensity continuous training (MICT) amplifies the IGF-1/PI3K/AKT cascade, driving robust phosphorylation and nuclear export of FoxO1 (Hong et al., 2025). High-intensity interval training (HIIT), by contrast, blunts that anabolic signaling and even raises FoxO1 expression through greater oxidative stress. The outcome is that MICT favors protein preservation, whereas HIIT may tilt toward transient catabolism that later triggers mitochondrial biogenesis. These rodent findings mirror clinical anecdotes in frail or sarcopenic adults, where overly aggressive intervals can cost lean tissue before conferring metabolic gains, underscoring the need for graded progression.

A single bout of resistance exercise in healthy adults lowers FoxO3a mRNA within 4 h, signalling relief from proteolysis. The superimposition of mild local heat further boosts MYOD1, a surrogate for myogenic commitment (McGlynn et al., 2024). The thermal potentiation hints that peripheral temperature is an under-appreciated modulator of FoxO-mediated repair, clinically relevant for postoperative or immobilized limbs that often remain cooler than active muscle.

Twelve weeks of eccentric-biased training in untrained men yields larger basal phosphorylation of both FoxO1 and FoxO3a than concentric training (Stefanetti et al., 2014). That sustained inactivation curtails Atrogin-1/MuRF1 transcription and aligns with the superior hypertrophy commonly observed after eccentric loading. Notably, adjunct whey hydrolysate failed to add further FoxO suppression, indicating that mechanical tension, not substrate provision, is the dominant cue for these transcription factors in young muscle.

An acute lumbar extension session in patients undergoing spinal surgery still depresses FoxO3a. However, the expected rise in myogenic transcripts is absent; instead, genes related to inflammation and extracellular matrix remodeling surge (Shahidi et al., 2024). The data imply that the FoxO switch is necessary for diseased muscle but is insufficient. Additional blockers of sterile inflammation or fibrosis may be prerequisites for effective regeneration.

In rats, only 7 days of inactivity after HIIT doubles FoxO3a expression while NF-κB falls, and MuRF1 rebounds in step with FoxO3a, not NF-κB (Sheibani et al., 2024). The finding refines our mechanistic view. During unloading, the FoxO3a/MuRF1 axis is the principal driver of atrophy, whereas NF-κB plays a secondary, perhaps inflammation-limited, role. Clinically, this warns that even short rehabilitation gaps can erase weeks of training gains.

Collectively, these studies reveal a unifying trend. Exercise suppresses FoxO-driven catabolism. Yet they also expose critical modifiers: aerobic intensity, contraction mechanics, thermal milieu, pathological status, and detraining all recalibrate which FoxO isoform is targeted and through what upstream pathway. Rodent’s work continues to map the signalling architecture, but the human data remains sparse and occasionally discordant. Therefore, Future trials must couple detailed FoxO phenotyping with clinically meaningful outcomes (strength, function, recovery) across diverse patient groups to close the translational gap and define an evidence-based amount of exercise that sustainably harnesses FoxO biology for muscle preservation and repair.

#### 3.3.2 Mitochondrial function and autophagy

Mitochondrial function and autophagy are essential cellular processes modulated by FoxO proteins in response to various exercise interventions. The following studies investigate how different exercise modalities and external interventions influence FoxO signaling, mitophagy, and mitochondrial dynamics (Supplementary Table S1).

Incremental-load swimming to exhaustion in young rats drives robust nuclear FoxO3 accumulation and upregulates the mitophagy’s GABARAPL1, PINK1/Parkin, and BNIP3, culminating in swollen, function-compromised mitochondria. Acute cannabidiol administration attenuates this surge, normalizing organelle morphology and respiratory capacity. This shows that FoxO3 can be therapeutically reduced when autophagic turnover increases (Si et al., 2024). By contrast, moderate treadmill running performed under hypoxia enhances Sirt3‐mediated deacetylation of FoxO3, thereby boosting SOD2 expression and PINK1/Parkin-dependent mitophagy; genetic or pharmacological Sirt3 blockade abrogates these benefits, underscoring a Sirt3/FoxO3 axis that is necessary for efficient renewal of the mitochondrial pool when oxygen supply is limited (Ma et al., 2022).

A single bout of unilateral resistance exercise or work-matched cycling in healthy young men elicits rapid FoxO3 nuclear import within 60 min. Yet, the downstream axis diverges. Resistance exercise sustains LC3-II turnover and p62 clearance for at least 4 h. In contrast, aerobic exercise (AE) shows an earlier return toward baseline, implying that mechanical tension amplifies and prolongs FoxO-driven autophagy (Mazo et al., 2021). The scenario shifts again in middle-aged men with type 2 diabetes mellitus (T2DM). During identical aerobic exercise, FoxO3 is phosphorylated on Ser253, suggesting partial cytoplasmic retention, but ULK1-Ser555 phosphorylation and LC3-II depletion still proceed, indicating that alternative cues (e.g., AMPK) can maintain autophagic flux when FoxO3 is restrained. Mitochondrial fission responses are preserved, whereas fusion is blunted, highlighting a disease-specific uncoupling between quality control and network remodeling (Kruse et al., 2017).

Taken together, these studies reveal that the direction of FoxO3 signalling is highly context-dependent. Hypoxia or mechanical overload require FoxO3 activation to clear damaged mitochondria and bolster antioxidant defenses, whereas extreme metabolic stress (exhaustive swimming) benefits from tempering the same pathway. Upstream regulators also differ. Sirt3 dominates under hypoxia, Akt under nutrient-replete recovery, and cannabinoid-sensitive kinases during exhaustive stress. This provides multiple therapeutic entry points. Notably, the human data demonstrate that exercise mode dictates the magnitude and persistence of FoxO3-autophagy coupling, and metabolic disease can selectively mute FoxO3 nuclear function without abolishing autophagy, implying compensatory activation of parallel axes such as ULK1/AMPK.

Although rodent work maps the mechanistic landscape, human evidence remains limited to acute studies in small cohorts. Whether chronic modulation of the Sirt3/FoxO3 node can restore mitochondrial health in T2DM, sarcopenia, or statin myopathy is unknown. Likewise, the cannabidiol findings warrant cautious exploration in clinical trials, given the balancing act between insufficient and excessive mitophagy. Finally, head-to-head comparisons of resistance versus aerobic training are needed to define the optimal stimulus for FoxO-mediated mitochondrial remodeling.

#### 3.3.3 Signaling pathways and molecular markers

The regulation of FoxO proteins under different exercise conditions plays a critical role in muscle maintenance, adaptation, and degeneration. This section summarizes studies that explore how various training regimens and physiological conditions influence FoxO pathways, oxidative stress, muscle atrophy, and antioxidant mechanisms (Supplementary Table S1).

Lifelong moderate-to-high-intensity treadmill running in old rats keeps FoxO1 phosphorylated and cytoplasmic, paralleling robust Akt/mTOR signalling and strong antioxidant defenses. When these animals are subsequently detrained for 8 months, FoxO1 becomes dephosphorylated and nuclear, Keap1 rises, Nrf2 falls, and biochemical markers of ferroptosis surge. These changes are far more pronounced in fast-glycolytic than slow-oxidative fibers (Wang ZZ. et al., 2023). The same PI3K/Akt axis that safeguards protein during chronic loading reveals a fiber-type hierarchy in susceptibility to inactivity-induced redox stress.

A single downhill-running bout in young rats acutely elevates FoxO1 mRNA in the fast vastus lateralis but not in the slow soleus. In contrast, after adaptation is complete, 6 weeks of repeated eccentric sessions ultimately suppress FoxO1 in the vastus (Azad et al., 2016). The transient upregulation drives a fast-to-slow myofiber shift (greater MHC I/IIa/IIx, lower IIb), indicating that FoxO1 participates in early remodeling rather than long-term atrophy during unaccustomed mechanical strain. The soleus, in turn, reduces several MHC isoforms without altering FoxO1, underscoring muscle-specific regulatory axis that cannot be extrapolated across fiber classes.

When young rats are fasted for 24 h but perform light treadmill running, fasting alone dephosphorylates FoxO1 (Ser^256^) and blunts Akt/mTORC2. The addition of mild exercise restores Akt activity, rephosphorylates FoxO1, and elevates local T3 through deiodinase-2 induction (Giacco et al., 2022). The nutrient-exercise interaction shows that even low mechanical stress can override fasting-induced catabolic signalling by modulating insulin-like and thyroid-hormone pathways.

In older humans who completed 12 weeks of whole-body resistance training, phosphorylated FoxO3a increases while total FoxO1 falls, and cannabinoid-receptor-1 (CB1) protein unexpectedly rises (Dalle and Koppo, 2021). The CB1 surge correlates with MyoD and Pax7, hinting that endocannabinoid signalling may help restrain FoxO1 while promoting myogenic repair in ageing muscle. Human data suggest that resistance exercise tends to suppress, rather than activate, FoxO-driven catabolism, contrasting with the acute catabolic signature sometimes seen in rodent eccentric paradigms.

These findings demonstrate that FoxO activation is context-dependent rather than an obligatory outcome of exercise. Nuclear FoxO typically rises when contractile load is abruptly withdrawn (detraining), when muscles face unaccustomed mechanical strain (acute eccentric work), or when metabolic stress is unbuffered (fasting without exercise). Chronic loading—whether endurance or resistance—usually promotes FoxO inactivation, provided upstream Akt and hormonal cues remain intact. Pronounced species and fiber-type differences emerge. Rodent fast muscles show larger FoxO swings than slow ones, whereas the limited human evidence points to overall FoxO suppression during resistance training. Thus, FoxO signalling is a regulator that integrates load history, fiber phenotype, age, endocrine status, and even endocannabinoid tone to regulate muscle proteostasis.

Across exercise modes and physiological conditions, FoxO proteins emerge as load—and metabolism-sensitive transcription factors whose phosphorylation status orchestrates muscle remodeling, oxidative defense, and catabolism. Divergent responses among species and fibre types highlight the need for mechanistic studies that couple molecular readouts with functional endpoints in humans. Closing this gap will clarify when FoxO inhibition is beneficial and when transient activation supports adaptive fiber transitions, ultimately guiding exercise prescriptions and adjunct therapies for muscle preservation.

#### 3.3.4 Muscle remodeling, growth, and adaptation

This section reviews various studies investigating the regulation of muscle remodeling, growth, and adaptation through FoxO proteins under different conditions, including exercise, supplementation, and aging. The findings highlight the complex interplay between catabolic and anabolic pathways and the potential for specific interventions to modulate these processes (Supplementary Table S1).

A single-leg resistance bout and protein intake lowered basal FoxO1/3a content in early-postmenopausal women when preceded by red-clover isoflavones. Yet, the acute phosphorylation pattern and anabolic signalling (p-Akt, p-mTOR) after exercise were identical to placebo (Oxfeldt et al., 2020). By contrast, brief cold-water immersion (10°C, 15 min) after an equivalent resistance session suppressed the exercise-induced fall in FoxO3a protein and attenuated myofiber hypertrophy despite unchanged strength gains, indicating that cooling sustains a catabolic milieu that selectively limits growth (Peake et al., 2020; Fyfe et al., 2019). In sedentary and non-smoking older adults completing 12 weeks of low-load elastic-band training, repeated FoxO3 downregulation paralleled gains in lean mass and a fast-to-intermediate fibre shift, underscoring that even modest loading can quell FoxO-driven proteolysis when recovery is thermoneutral (Krause et al., 2019).

During moderate treadmill running, carbohydrate ingestion boosted Akt phosphorylation and thereby maintained FoxO1/3a in an inactive state, reducing MURF1 and Atrogin-1 expression and net protein breakdown in recreationally active men (Margolis et al., 2018). In older women, however, 12 weeks of progressive cycling lowered FoxO3A mRNA by ∼24% without altering protein phosphorylation, hinting at transcriptional, not post-translational, suppression as a mechanism compatible with age-related anabolic resistance (Konopka et al., 2010). In REDD1-knock-out mice, where mTORC1 is chronically hyperactive, an acute treadmill bout failed to elevate FoxO1/3a phosphorylation, illustrating that upstream energy-stress sensors (AMPK, REDD1) gate FoxO responsiveness to endurance stimuli (Dungan et al., 2019).

Not all decreases in phosphorylation are harmful. In mdx mice, high-intensity running dephosphorylated FoxO1/3a, releasing autophagy that lessened fibre necrosis and improved regeneration (Zhou et al., 2024). Similarly, lifespan-long voluntary running in aged rats increased *phosphorylated* FoxO1 but simultaneously elevated antioxidant enzyme SOD2, implying that periodic FoxO cycling, rather than constitutive inactivation, optimizes redox homeostasis during ageing (Gao et al., 2020). These rodent data caution against a blanket strategy of maximal FoxO suppression in clinical settings.

Post-exercise blends of soy protein, amylopectin, and chromium in rats curtailed FoxO1/3a protein and ubiquitin-proteasome activity while amplifying IGF-1/Akt/mTOR signalling, yielding the greatest hypertrophic response when combined with exercise (Kayri et al., 2019). Similar reductions in FoxO protein occur in humans given red-clover extract but without measurable hypertrophy over 2 weeks (Oxfeldt et al., 2020), highlighting a common molecular signature.

Human studies overwhelmingly involve short-term interventions (≤12 weeks) and healthy volunteers, whereas mechanistic depth remains confined to rodents. Significantly, pharmacological FoxO modulators effective in rats or mice have yet to enter human trials, marking a clear translational gap.

Current evidence supports a model in which acute phosphorylation-dependent *inactivation* of FoxO underpins muscle recovery and hypertrophy after resistance and endurance exercise in humans, provided recovery conditions do not re-activate the pathway. Yet rodent data reveal circumstances where transient FoxO *activation* promotes autophagy, redox balance, and tissue preservation. Therefore, rather than pursuing wholesale inhibition, future human studies should aim to accommodate the *timing, amplitude, and duration* of FoxO signalling according to exercise mode, nutritional state, and clinical phenotype. Defining these context-specific FoxOs is essential for translating molecular insights into targeted therapies.

### 3.4 Muscle-specific studies: recovery and hypertrophy

This section examines muscle-specific studies focused on recovery and hypertrophy, particularly involving FOXO protein regulation under different exercise protocols and supplementation strategies. These findings emphasize the modulation of catabolic and anabolic pathways to enhance muscle growth and recovery (Supplementary Table S1).

In aged soleus (a predominantly oxidative muscle) high-intensity interval training (HIIT) only blunted FoxO3 when it was paired with branched-chain amino acids (BCAA) carried in nano-chitosan; upslope running further amplified this effect, implying that both a nutrient signal (leucine-sensitive mTORC1) and the higher mechanical load typical of incline work are necessary to override the catabolic FoxO program in senescent slow-twitch fibers (Sadri et al., 2020). By contrast, in the younger, mixed-fiber gastrocnemius, the same HIIT stimulus without supplements suppressed FoxO1 and simultaneously raised Akt phosphorylation, lowered myostatin/Smad2-3 signaling, and expanded cross-sectional area (Biglari et al., 2020). Taken together, these data suggest that the fast/slow fiber composition and nutritional state dictate whether FoxO1 or FoxO3 is the dominant brake on hypertrophy, as well as whether Akt/mTOR or the myostatin/Smad pathway provides the primary counter-signal.

Resistance training introduces a different regulatory layer. When ladder-climbing in rats was combined with pharmacological TGF-β1/Smad blockade, FoxO1/3 transcripts fell more sharply than with loading alone, and mTOR/S6K1 activation increased commensurately (Nikooie et al., 2020). This synergy suggests crosstalk: both FoxOs and Smads converge on the promoters of atrogenes, such as MuRF1/Atrogin-1, so simultaneous suppression of each pathway may be required for maximal protein synthesis under high mechanical tension.

Following myocardial infarction, systemic inflammation and circulating catabolic cytokines typically dephosphorylate (activate) FoxO3, accelerating soleus wasting. Progressive resistance exercise partially restored FoxO3 phosphorylation (the inactive, cytoplasmic form), reinstated redox enzymes, and attenuated glycolytic over-reliance, thereby limiting proteolysis and preserving contractile architecture (Souza et al., 2023). The finding underscores that, in disease, the beneficial FoxO shift arises less from increased Akt signaling and more from the dampening of stress kinases that otherwise drive FoxO nuclear import.

Mechanistically, the studies converge on a unifying theme. FoxO inhibition is necessary but not sufficient for muscle growth. A second signal is always present: nutrient-dependent mTORC1 in the HIIT + BCAA model, load-induced S6K1 in resistance training, or redox normalization after infarction. The relative contribution of FoxO1 versus FoxO3 also appears to be fiber-type dependent, with FoxO3 dominating in oxidative muscles and FoxO1 shaping responses in glycolytic or mixed fibers.

Collectively, these muscle-specific studies show that FoxO proteins act as an adaptable regulator rather than a simple on/off switch for hypertrophy. Exercise intensity, contraction mode, muscle phenotype, nutritional cues, and pathological stress each bias the regulatory role differently, explaining why identical changes in FoxO phosphorylation can lead to divergent outcomes. Integrating FoxO modulation with the parallel Akt/mTOR, myostatin/Smad, and redox axes, therefore, offers a more precise blueprint for designing exercise or adjuvant therapies that maximize recovery and growth while minimizing atrophy in both healthy and diseased states.

## 4 Impact of exercise on FoxO pathway in cardiac muscle

In this section, we discuss how different exercises influence the FoxO signaling pathway in cardiac muscle, focusing on the pathways through which physical activity confers protective or adaptive responses in the heart. We have summarized the key findings from these studies in Table 1.

**TABLE 1:** Overview of selected studies examining the influence of various exercise protocols on the FoxO signaling pathway in cardiac muscle. Each entry highlights the exercise type, duration, experimental groups, changes in FoxO regulation, and principal findings related to cardiac health and function.

Exercise type	Samples	FoxO regulation by exercise	Main findings	Refs
Myocardial infarction				
Moderate and prolonged exercise training	47 male rats	↑ FoxO1 expression (activation)	Moderate, prolonged exercise increases SIRT3 expression, activating the FoxO3a pathway. Enhanced MnSOD and catalase expression reduces oxidative stress. SIRT1 inhibition reduces the exercise-induced antioxidant effect. Exercise training reduces cardiomyocyte apoptosis, especially in the infarct border zone, promoting cardioprotection.	Donniacuo et al. (2019)
Moderate Intensity interval training performed on a treadmill	25 male rats (12 weeks old)	↑ FoxO3a phosphorylation (inactivation)	Exercise training and taurine reduce FoxO3a and Caspase-8 activity, enhancing Akt activation. Improved cardiac function (SV, EF, FS) and reduced infarct size. Combination of both interventions more effective than either alone. Reduced collagen deposition and myocardial necrosis.	Razzaghi et al. (2023)
Aerobic treadmill running	Rats (8-week-old)	Stabilized FoxO3a mRNA (activation)	Exercise training reduced WTAP expression and m6A modification of FOXO3a mRNA, enhancing cardioprotection by reducing apoptosis and inflammation in I/R injury. WTAP/YTHDF1/m6A/FOXO3a axis is key in myocardial I/R injury progression	Wang et al. (2023b)
Swimming exercise	Male mice (8–10 weeks)	↓ FoxO1 expression (inactivation)	miR-486 inhibits FoxO1 and PTEN, activating AKT/mTOR pathways. Enhanced cardiomyocyte survival, reduced apoptosis. Exercise-induced miR-486 protects against cardiac I/R injury. miR-486 deficiency diminishes exercise’s cardioprotective effects.	Bei et al. (2022)
Cardiomyopathies and Coronary Heart Disease				
High-intensity interval training for humans and treadmill running for mice​	Humans: 49 obese men (20–60 years) Mice: Healthy mice, FGF21 KO mice, hepatocyte-selective FGF21 KO mice​	↑ FoxO3a phosphorylation (inactivation)	Exercise activates the AMPK/FOXO3/SIRT3 pathway, enhancing mitochondrial integrity and reducing oxidative stress in the heart. FGF21-induced activation of FOXO3 phosphorylation is crucial for the exercise-mediated cardioprotective effects against diabetic cardiomyopathy.	Jin et al. (2022)
High-Intensity Interval training, Moderate-intensity continuous training	35 male rats (8-weeks-old)	↑ FoxO3a phosphorylation (inactivation)	Exercise and quercetin regulate the PI3K/AKT/FOXO3 pathway, reduce FOXO3 nuclear translocation, inhibit apoptosis, and improve lipid profiles and inflammatory markers.	Khajehlandi and Bolboli (2025)
Progressive endurance treadmill training	female LC3 transgenic and healthy mice (6-month-old)	↑ FoxO1 phosphorylation (inactivation)	Exercise preconditioning preserved phosphorylated FOXO1 levels, preventing FOXO1 activation. It mitigated muscle atrophy and metabolic dysfunction in tumor-bearing mice. FOXO1 signaling was modulated to maintain beneficial autophagy.	Parry et al. (2022)
Moderate-intensity aerobic exercise (treadmill)	165 patients with stable coronary heart disease	↓ FoxO3a phosphorylation (inactivation)	Exercise activates the FoxO3a-Sirt6 axis via EETs, reduces sEH activity, and decreases PCSK9 expression, enhancing LDLR expression and lowering LDL-C levels in CHD patients and hyperlipidemic mice.	Hu et al. (2025)
Cardiac Metabolism and Angiogenesis				
Swimming with progressively increased load resistance	Male rats (7 weeks old)	↑ FoxO1 expression (activation)	Exercise increases FOXO1 protein levels post-transcriptionally via miR-223-3p downregulation, enhancing GLUT4 expression and glucose uptake.	Carrillo et al. (2023)
Low-intensity treadmill running	27 Male Wistar rats	↑ FoxO1 nuclear deacetylation (activation)	Low-intensity exercise increased nuclear FOXO1 and SIRT1 levels, promoting fatty acid oxidation. FOXO1 deacetylation by SIRT1 enhances transcriptional activity, supporting metabolic adaptation. Fructose-fed rats benefited from exercise through improved FOXO1 regulation and cardiac energy metabolism	Kostić et al. (2023)
Treadmill Exercise	35 male rats	↓ FoxO1 expression (inactivation)	Exercise and sodium butyrate improved angiogenesis by reducing miR-34a, FOXO1 expression, enhancing SIRT1, and HIF-1α levels. Combined treatment showed the most significant improvement in diabetic heart tissue, suggesting therapeutic potential via the miR-34a/SIRT1/FOXO1-HIF-1α pathway.	Sarlak et al. (2023)
Physiological and Pathophysiological Cardiac Changes				
Swimming Exercise (aerobic exercise)	40 Spontaneously hypertensive rats and Wistar Kyoto rats (12-week-old)	↑ FoxO3 nuclear deacetylation (activation)	Exercise and DF peptide synergistically activate AMPK/SIRT1/PGC-1α/FOXO3, enhancing mitochondrial biogenesis, reducing apoptosis, hypertrophy, and fibrosis in hypertensive rats.	Ho et al. (2022)
Swimming Exercise (aerobic exercise)	40 Spontaneously hypertensive rats and Wistar Kyoto rats	↑ FoxO3a phosphorylation (inactivation)	Relationship between exercise/training/activity and FOXO proteins: Exercise training combined with VH-4 peptide treatment increased the expression of FoX3α through AMPKα1, Sirt1, and PGC1α signaling pathways, improving cell survival and reducing hypertension-induced damage.	Baskaran et al. (2022)
Swimming Exercise (aerobic exercise)	24 rats (18 months old)	↑ FoxO3 phosphorylation (inactivation)	Exercise activates PI3K-Akt, phosphorylating FOXO3 and retaining it in the cytoplasm. Resveratrol activates SIRT1, promoting FOXO3 deacetylation. Combined treatment blocks FOXO3 nuclear accumulation, reduces apoptosis, and improves heart function in aged rats.	Lin et al. (2014)
Progressive swim training	Female mice (aged 8–12 weeks)	↑ FoxO1 phosphorylation (inactivation)	Exercise-induced hypertrophy requires FoxO1. FoxO1 deletion attenuates hypertrophy and reduces Hsp70 expression. Autophagy regulation by FoxO1 contributes to hypertrophic response.	Weeks et al. (2021)

↑ increased; ↓ decreased.

### 4.1 Cardiovascular disease and injury mechanisms

#### 4.1.1 Myocardial infarction

Myocardial infarction (MI) triggers a surge of oxidative stress, inflammatory signaling, and apoptotic loss of cardiomyocytes. Four recent rodent studies demonstrate that exercise intersects the FoxO network at several experimentally distinct nodes, and the direction of FoxO regulation determines the outcome (Table 1).

In Sprague–Dawley rats, 8 weeks of moderate treadmill running upregulated the mitochondrial deacetylase SIRT3; the ensuing deacetylation and nuclear retention of FoxO3a amplified the transcription of manganese superoxide dismutase and catalase, lowered reactive oxygen species in the peri-infarct border zone, and limited early-phase apoptosis (Donniacuo et al., 2019). By contrast, when the same exercise paradigm was combined with taurine, the dominant upstream signal shifted to the PI3K/AKT pathway. AKT-mediated phosphorylation of FoxO3a at Thr^32^/Ser^253^ expelled the factor from the nucleus, suppressing the pro-apoptotic gene Casp8, halving infarct size, and normalizing stroke volume and ejection fraction (Razzaghi et al., 2023).

A third mechanistic layer involves post-transcriptional control. Myocardial ischemia–reperfusion (I/R) injury elevates the m6A “writer” WTAP; increased methylation stabilizes Foxo3a mRNA, driving over-expression of FoxO3a and worsening I/R injury. Six weeks of treadmill running downregulated WTAP and YTHDF1, attenuating FoxO3a accumulation, dampening inflammatory cytokines, and improving histological repair (Wang H. et al., 2023). Finally, swimming exercise in mice enhanced cardioprotective miR-486, which simultaneously repressed Foxo1 and the phosphatase PTEN, thereby activating AKT/mTOR survival signaling and further reducing apoptosis after I/R (Bei et al., 2022).

Across these studies, two themes emerge. First, the same FoxO isoform can be either cardioprotective or cardiotoxic depending on the upstream milieu. When SIRT3 is dominant, nuclear FoxO3a favors an antioxidant transcriptional program; when AKT is dominant, cytosolic (phosphorylated) FoxO3a prevents the transcription of pro-death genes. Second, the timing of FoxO modulation appears critical. Nuclear activation early after MI is beneficial for redox control, whereas sustained activation later in the remodeling phase promotes maladaptive apoptosis and fibrosis. Species (mouse vs. rat), exercise modality (treadmill vs. swimming), and adjunctive factors (taurine, m6A flux, microRNAs) further fine-tune the balance between activation and repression, underscoring why apparently redundant observations of FoxO phosphorylation can translate into opposite physiological outcomes.

#### 4.1.2 Cardiomyopathies and coronary heart disease

This section presents recent studies focusing on the regulation of FoxO proteins through various exercise-induced pathways and their implications for cardiomyopathies and coronary heart disease.

In murine models of diabetic cardiomyopathy, high-intensity treadmill running activates an FGF21/AMPK/SIRT3 cascade that phosphorylates FoxO3, stabilizes mitochondria, and limits oxidative injury (Jin et al., 2022). Because FGF21 biology differs between rodents and humans, future trials will need to verify whether circulating FGF21 or its hepatic release is the dominant AMPK stimulus in people with diabetes.

When male rats performed either high-intensity interval training (HIIT) or moderate continuous training while receiving quercetin, the nutrient–exercise combination amplified PI3K/AKT signaling, blocked FoxO3 nuclear entry, and lowered apoptosis, dyslipidemia, and inflammation (Khajehlandi and Bolboli, 2025). This study demonstrates that bioactive compounds can alter the threshold at which exercise alone modulates FoxO; however, the absence of female animals and the quercetin dose complicate direct clinical extrapolation.

A contrasting scenario is cancer-induced cardiac wasting, where 6 weeks of progressive endurance running in female tumor-bearing mice increased FoxO1 phosphorylation, thereby sustaining adaptive autophagy while preventing maladaptive remodeling (Parry et al., 2022). These data emphasize isoform-specific control: FoxO1 appears to govern cachexia-related metabolism, whereas FoxO3 predominates in metabolic cardiomyopathies.

Human evidence, although still limited, supports the translational relevance of these mechanisms. In 165 patients with stable coronary heart disease, moderate aerobic training enhanced epoxy-eicosatrienoic acids, activated the FoxO3a/Sirt6 axis, suppressed PCSK9, and ultimately lowered LDL-C despite ongoing statin therapy (Hu et al., 2025). Notably, FoxO3 phosphorylation decreased in blood cells, even though cardiac tissue was not sampled, suggesting that easily accessible surrogates may track myocardial FoxO activity in future trials.

Across models, the phosphorylation-dependent inactivation of FoxO proteins emerges as a unifying cardioprotective signature. Yet, the upstream cues (AMPK versus AKT versus eicosanoid signalling), the dominant FoxO isoform (FoxO1 versus FoxO3), and the pathological drivers (hyperglycemia, inflammation, tumor burden, hyperlipidemia) differ. These distinctions explain why identical read-outs, such as reduced apoptosis or improved lipid handling, arise from mechanistically diverse interventions. They also highlight gaps. Collectively, the current literature demonstrates that exercise redirects maladaptive cardiac signaling toward survival pathways by converging on FoxO phosphorylation; however, the route to that convergence is context-specific. Clarifying which upstream nodes are most druggable in humans—AMPK, PI3K/AKT, or lipid-derived mediators—and determining how training variables (intensity, modality, and concomitant nutrition) influence those nodes will be essential for converting this mechanistic insight into targeted therapies for cardiomyopathies and coronary heart disease.

### 4.2 Cardiac metabolism and angiogenesis

Recent research highlights the role of exercise in enhancing cardiac metabolic efficiency and promoting angiogenesis through the modulation of FoxO proteins (Table 1).

In healthy juvenile rats exposed to progressively heavier swim sessions, a sharp decline in miR-223-3p relieves post-transcriptional repression of FoxO1, allowing nuclear accumulation that drives GLUT4 transcription and accelerates cardiac glucose clearance (Carrillo et al., 2023). In fructose-fed Wistar rats performing low-intensity treadmill running, FoxO1 not only rises in abundance but is enzymatically reset. SIRT1 removes inhibitory acetyl groups, allowing FoxO1 to shift its focus toward genes that favor fatty acid oxidation over lipid storage (Kostić et al., 2023). Although both activate FoxO1, they rely on different molecular levers—microRNA suppression versus deacetylation—and their metabolic outcomes diverge in substrate preference (glucose versus fatty acids). This emphasizes that FoxO1 activity is finely graded rather than simply switched on or off.

A different picture emerges in the diabetic myocardium. Combining moderate treadmill training with the histone-deacetylase metabolite sodium butyrate lowers miR-34a, reduces FoxO1 protein, and permits a HIF-1α-driven angiogenic pathway to dominate (Sarlak et al., 2023). Suppressing FoxO1 is advantageous here because excessive FoxO1 can oppose HIF-1α-mediated vessel formation and exacerbate oxidative stress. Thus, when oxygen delivery is the critical constraint, dampening FoxO1 becomes cardioprotective.

Taken together, these rodent studies show that both the direction (up- or downregulation) and the qualitative nature (post-transcriptional versus post-translational) of FoxO1 control match the dominant stressor—substrate overload, lipotoxicity, or hypoxia.

### 4.3 Physiological and pathophysiological cardiac changes

Recent research demonstrates the beneficial modulation of cardiac function through various signaling pathways involving FoxO proteins (Table 1).

Two hypertensive rat studies illustrate the cardioprotective face of FoxO3 activation. In spontaneously hypertensive rats, treadmill exercise combined with the DIKTNKPVIF peptide enhanced AMPK and SIRT1 activity, leading to FoxO3 deacetylation, mitochondrial biogenesis, and a coordinated reduction in oxidative stress, apoptosis, and fibrosis (Ho et al., 2022). A parallel study substituting the soybean peptide VH-4 for DIKTNKPVIF produced a closely aligned AMPKα1/SIRT1/PGC-1α response, again lowering hypertensive damage, despite reporting FoxO3 phosphorylation rather than deacetylation (Baskaran et al., 2022). The mechanistic convergence—improved mitochondrial quality control—underscores that post-translational status (phosphorylated vs. deacetylated) can be less important than the broader signaling context in which FoxO3 operates.

In aged rat hearts, endurance swimming alone phosphorylated FoxO3 via the PI3K/Akt pathway, favoring its cytoplasmic retention. Adding the SIRT1 activator resveratrol tipped the balance back toward nuclear FoxO3 exclusion without abolishing AMPK signaling. The dual intervention minimized apoptosis and fibrosis while improving diastolic function (Lin et al., 2014). This experiment illustrates how FoxO3 “inactivation” can still be beneficial when it prevents the persistence of pro-apoptotic gene programs that dominate in senescence.

Progressive swim training in adult female mice induced canonical physiological hypertrophy; the response was blunted when FoxO1 was deleted. Although Akt-mediated FoxO1 phosphorylation nominally reduces its transcriptional activity, residual nuclear FoxO1 proved indispensable for upregulating autophagy genes and the chaperone Hsp70, both of which are critical for protein quality control during growth. Thus, phosphorylation is an oversimplification; low-level FoxO1 activity appears permissive for adaptive remodeling (Weeks et al., 2021).

Across these rodent models, aerobic exercise was the standard stimulus. Whether resistance training or mixed-modality exercises, more typical of human cardiac rehabilitation, evoke the same FoxO patterns remains untested. Species differences also matter: rodent hearts rely more heavily on glycolytic flux and manifest larger Akt responses than adult human hearts, potentially magnifying FoxO phosphorylation. Limited human data (endomyocardial biopsies from endurance athletes and explanted failing hearts before/after ventricular unloading) suggest exercise reduces nuclear FoxO3 abundance and increases PGC-1α expression, but sample sizes are small, and longitudinal evidence is lacking. Moreover, nutraceutical co-interventions (DIKTNKPVIF, VH-4, resveratrol) that accentuated FoxO signaling in rodents have not been evaluated clinically, highlighting a clear translational gap.

## 5 Comparison of FoxO regulation in skeletal vs. cardiac muscle

This comparative section places the principal findings for skeletal and cardiac muscle in parallel, highlighting both convergences and divergences in FoxO signaling during exercise (Table 2). In skeletal muscle, research predominantly centers on FoxO3a-mediated proteolysis; endurance training and, especially, resistance exercise commonly phosphorylate or acetylate FoxO3a, thereby reducing MuRF1 and Atrogin-1 expression and limiting atrophy. Cardiac studies distribute attention between FoxO3a and FoxO1 and focus on oxidative stress, apoptosis, and metabolic adaptation. In the heart, exercise may promote nuclear, deacetylated FoxO3a to enhance antioxidant defenses shortly after injury, yet drive phosphorylated (cytoplasmic) FoxO isoforms during later remodeling to restrain cell death. Up-stream regulators differ accordingly; skeletal muscle paradigms emphasize mechanical and metabolic checkpoints such as HDAC4 and Sestrin2, whereas cardiac investigations highlight endocrine and epigenetic inputs, including FGF21, microRNAs, and m6A-dependent mechanisms. Despite these context-specific pathways, a common principle emerges. Exercise re-directs FoxO activity toward protective outcomes when the training modality is matched to the predominant upstream signal in each tissue and disease state.

**TABLE 2:** Key features of exercise-induced FoxO signalling in skeletal versus cardiac muscle. The matrix contrasts the dominant FoxO isoforms, upstream regulators, direction of modulation, downstream gene programs, functional outcomes, contextual modifiers, and clinical implications for each tissue.

Dimension	Skeletal muscle	Cardiac muscle	Distinguishing points
Predominant FoxO isoforms	FoxO3a is explored more than other FoxO isoforms especially FoxO1.	Both of FoxO3a and FoxO1 are explored. However, isoform dominance flips with the context.	Skeletal work focuses on proteolytic FoxO3a; cardiac studies split between FoxO3a (oxidative stress) and FoxO1 (metabolism, hypertrophy).
Typical exercise modalities	Endurance (treadmill, wheel) Resistance/overload (ladder, weights) Mixed or HIIT	Endurance (treadmill, swimming) HIIT (metabolic cardiomyopathy)	Resistance experiments dominate in skeletal muscle; cardiac literature is almost entirely aerobic or interval-based.
Up-stream regulators recruited by exercise	AKT, AMPK, SIRT1/2/3, HDAC4, Sestrin2, PGC-1α, NF-κB, MSTN/Smad	AKT, AMPK, SIRT1/3/6, FGF21, eicosanoids, miRNAs (miR-486, miR-34a, miR-223-3p), m6A modification (WTAP), peptides (VH-4, DF)	Cardiac studies emphasize endocrine or epigenetic inputs; skeletal work highlights local mechanical/metabolic brakes (HDAC4, Sestrin2).
Direction of FoxO modulation produced by exercise	Mostly inhibitory (↑Ser^253^/Thr^32^ phosphorylation or ↑acetylation → cytoplasmic retention); a few models use constructive activation for mitophagy/antioxidant defense in ageing	Biphasic: Early/antioxidant benefit – deacetylated nuclear FoxO3a (via SIRT3) Chronic protection – phosphorylated cytoplasmic FoxO3/1 (via AKT/AMPK)	Cardiac outcome depends more on timing (acute vs. chronic) than absolute direction; skeletal outcome tracks direction more linearly (phosphorylation = anti-atrophy).
Key downstream axes affected	MuRF1/Atrogin-1 ubiquitin-proteasome axis Autophagy (LC3, BNIP3, Beclin-1) mTORC1 anabolic rescue	Antioxidant enzymes (MnSOD, catalase) Apoptosis regulators (Bax, Casp8) Metabolic genes (GLUT4, FA-oxidation) Angiogenesis (HIF-1α)	Proteostasis dominates skeletal focus; redox, survival, and metabolic remodeling dominate cardiac focus.
Functional/phenotypic outcomes	Preservation of mass, reversal of drug- or disease-induced atrophy, hypertrophy with resistance	Smaller infarct size, improved EF/SV, anti-fibrotic remodeling, physiological hypertrophy, better metabolic flexibility	Both tissues gain stress resistance, but endpoints differ (size/strength vs. function/survival).
Contextual modifiers highlighted	Pharmacologic catabolites (dexamethasone, cisplatin, doxorubicin, dapagliflozin), CKD, diabetes, hypoxia, ageing	Myocardial infarction, diabetic cardiomyopathy, hypertension, I/R injury, tumor cachexia, ageing, dyslipidemia	Cardiac studies integrate systemic diseases more; skeletal set explores therapy-induced wasting.
Conclusion	Exercise suppresses FoxO-driven proteolysis; modality choice (resistance vs. endurance) should target drug- or disease-specific upstream inhibitors.	Exercise re-balances FoxO between antioxidant defense and apoptosis control; intensity and adjuncts (taurine, resveratrol, eicosanoids) determine whether FoxO is held inside or outside the nucleus.	-

## 6 Neurological and cognitive benefits of exercise through FoxO pathways

In this section we have shown how FoxO signaling mediates the effects of exercise on brain function and cognitive performance. The data summarized in Table 3 demonstrates that different forms of physical activity, ranging from aerobic to resistance training, elicit changes in FoxO regulation, which in turn influence neuronal survival, mitochondrial function, and pathways associated with aging and neurodegenerative diseases.

**TABLE 3:** Impact of exercise on FoxO signaling within the nervous system. Each study highlights how various exercise modalities modulate FoxO pathways to promote neuroprotection, cognitive function, and improved neural health.

Exercise type	Samples	FoxO regulation by exercise	Main findings	Refs
Cognitive impairment and neuroprotection				
Aerobic exercise using a treadmill	42 participants	↑ FoxO1 expression (activation)	Exercise increased FOXO1 and FBXO32 expression in males with MCI but not females, indicating gender-dependent differences. Elevated FOXO1 promotes cellular clearance systems and neuroprotection, potentially mitigating Alzheimer’s progression by enhancing UPS and autophagy.	Bedada et al. (2021)
Aerobic Exercise (Swimming)	84 mice	FoxO3a activation (activation)	Exercise/irisin reduces cognitive impairment by enhancing Klotho, FOXO3a, MnSOD, and lowering ROS in cerebral ischemia. Klotho knockout nullifies these effects.	Jin et al. (2021)
High-intensity interval training	28 male rats	↑ FoxO3 nuclear deacetylation (activation)	HIIT enhances FOXO3 via lactate-induced SIRT1 activation. Increases PINK1/Parkin pathway activation. Improves mitophagy. Reduces Tau and Aβ accumulation in hippocampus. Mitigates Type 2 Diabetes-induced cognitive impairment.	Khosravi et al. (2024)
Aerobic exercise (treadmill running)	102 male rats	↑ FoxO1 phosphorylation (inactivation)	Aerobic exercise potentially inhibits FOXO1 by increasing phosphorylation, reducing acetylation, and suppressing NF-κB/NLRP3 inflammatory signaling in diabetic rats.	Wang et al. (2019)
Aerobic exercise on treadmill	42 Male mice (8 weeks-old)	↑ FoxO1 phosphorylation (inactivation)	Aerobic exercise downregulates DAPK1/CDKN2A/REDD1/FoxO1 signaling, reducing hippocampal apoptosis. Exercise enhances phosphorylation of FoxO1, inhibiting apoptosis via exogenous (Fas/FasL) and mitochondrial pathways. Improved cognitive performance observed in D-gal-induced aging mice following exercise.	Liu et al. (2023)
Neurodegenerative Diseases				
Treadmill Exercise	18 male mice	↑ FoxO1/3 nuclear deacetylation (activation)	Exercise upregulated the SIRT1-FOXO1/3 axis, improving mitophagy and reducing Aβ plaques in APP/PS1 mice. SIRT1 inhibition using EX527 reversed these positive effects, suggesting a crucial role of the SIRT1-FOXO1/3 axis in regulating mitophagy during exercise.	Zhao et al. (2023a)
Rotarod walking exercise	Male mice (7 weeks old)	↑ FoxO3a activation (inactivation)	Exercise and creatine enhance FoxO3a expression via SIRT3/FoxO3a signaling, resulting in antioxidant effects. Combined exercise and creatine produce additive effects on FoxO3a activation. Increased FoxO3a is associated with improved neuroprotection in the MPTP-induced PD model.	Leem et al. (2024)
Stroke and Ischemic Injury				
Treadmill running	54 Male mice (4–5 weeks-old)	↑ FoxO3a phosphorylation (inactivation)	Exercise pretreatment activates AMPK, enhancing FOXO3a activity, which promotes autophagic flux, reduces neuroinflammation, and mitigates oxidative stress.	Zhao et al. (2023b)
Treadmill running (a form of moderate aerobic exercise)	96 male rats	↑ FoxO1 phosphorylation (inactivation)	Exercise post-conditioning activates PI3K/AKT/FoxO1 signaling, enhancing phosphorylation of FoxO1, inhibiting gluconeogenesis, and reducing ischemic brain injury.	Li et al. (2023)
Miscellaneous: Muscle-Brain Crosstalk and Metabolic Control				
Progressive resistance exercise by climbing a ladder	22 male mice (3 months-old)	↑ FoxO phosphorylation (inactivation)	Resistance training enhances FoxO signaling in both brain and muscle. Upregulates Sgk1, which improves memory and synaptic plasticity. FoxO pathway alterations contribute to muscle-brain crosstalk.	Liu et al. (2024)
High Intensity Interval Training	20 male rats	↑ FoxO1 phosphorylation (inactivation)	High Intensity Interval Training increases leptin receptor expression, promoting JAK2 and STAT3 phosphorylation. Phosphorylated STAT3 regulates FoxO1 by phosphorylation, leading to its nuclear exclusion. High Intensity Interval Training decreases FoxO1 levels, enhancing POMC and CART expression, and reducing NPY and AGRP levels, improving appetite regulation	Khoramipour et al. (2023)

↑ increased; ↓ decreased.

### 6.1 Cognitive impairment and neuroprotection

Regular physical exercise has been shown to influence various molecular pathways associated with cognitive impairment and neuroprotection. This section presents recent studies that explore the relationship between exercise and protein regulation in cognitive health (Table 3).

A three-month aerobic training in African Americans with mild cognitive impairment (MCI) elevated skeletal-muscle and circulating FoxO1 and FBXO32 in men but not in women (Bedada et al., 2021). That sex-specific divergence, observable in the clinic rather than in pre-clinical models, underlines both the translational promise of the pathway (FoxO1 is inducible without pharmaceuticals) and the persistent knowledge gap (we do not know whether the same stimulus would modulate FoxO3a, or whether resistance or mixed-mode exercise might eliminate the female “non-responder” phenotype).

Pre-clinical studies elaborate the mechanistic palette through which different exercise modalities converge on FoxO signalling while still producing context-specific outcomes. In a mouse model of cerebral ischemia, voluntary wheel running increased myokine irisin; irisin, in turn, upregulated the longevity protein Klotho, permitting nuclear FoxO3a accumulation and MnSOD transcription, thereby quenching reactive oxygen species and improving cognition (Jin et al., 2021). By contrast, high-intensity interval training (HIIT) in diabetic rats relied on a metabolic cue—lactate—to activate SIRT1, deacetylate FoxO3, drive PINK1/Parkin-dependent mitophagy, and limit Tau and Aβ deposition (Khosravi et al., 2024). In another metabolic milieu (streptozotocin-induced diabetes), moderate-intensity treadmill running favored PI3K/AKT-mediated phosphorylation of FoxO1, keeping it out of the nucleus, thereby damping NF-κB/NLRP3 inflammasome activity and restoring synaptic plasticity (Wang et al., 2019). Finally, in aging mice, aerobic exercise reduced the expression of the pro-apoptotic DAPK1/CDKN2A/REDD1 axis while simultaneously phosphorylating and excluding FoxO1 from the nucleus, thereby curbing FasL-driven cell death and preserving memory (Liu et al., 2023). Taken together, these experiments demonstrate that FoxO activation (via deacetylation or nuclear import) is desirable when enhanced proteostasis or antioxidant defense is rate-limiting. In contrast, FoxO inhibition (via phosphorylation-dependent export) is beneficial when excessive catabolic or inflammatory signaling predominates.

A comparison highlights species and mode-of-exercise differences that the literature often glosses over. Rodent data are almost exclusively derived from treadmill or swimming paradigms that mimic steady-state endurance work, whereas the lone human trial used mixed aerobic machines; resistance training, known to produce qualitatively different PI3K/AKT and mTOR dynamics, remains virtually unexplored. Moreover, the molecular “switch” that toggles FoxO between protective and maladaptive roles varies across tissues or disease models. SIRT1-driven deacetylation predominates in diabetic HIIT, irisin-Klotho signaling in cerebral ischemia, and canonical PI3K/AKT phosphorylation in systemic inflammation. Whether these discrepancies reflect species-specific regulation, fiber-type composition, or simply the metabolic context (hyperglycemia, hypoxia, senescence) is unresolved.

### 6.2 Neurodegenerative diseases

In Na Zhao’s study, the FoxO protein is regulated through the activation of the SIRT1/FoxO1/3 axis following 12 weeks of treadmill exercise in APP/PS1 mice. This exercise regimen effectively enhanced PINK1/Parkin-mediated mitophagy activity in the hippocampus, thereby improving mitochondrial function and reducing β-amyloid plaque accumulation. The study demonstrated that SIRT1 activation, which is triggered by an elevated NAD+/NADH ratio, promotes the deacetylation and activation of FoxO1 and FoxO3, stabilizing PINK1 and facilitating mitophagy. Moreover, inhibition of SIRT1 using the specific inhibitor EX527 confirmed that the SIRT1-FoxO1/3 pathway is essential for exercise-induced mitophagy. This mechanism ultimately leads to ameliorated learning and memory deficits in Alzheimer’s disease mouse models, suggesting a potential therapeutic strategy targeting mitophagy dysfunction via the SIRT1-FOXO1/3 axis (Zhao N. et al., 2023).

In Yea-Hyun Leem’s study, the FoxO protein, specifically FoxO3a, is regulated through the SIRT3/FoxO3a signaling axis which is activated by creatine supplementation and exercise. This activation promotes antioxidative effects by enhancing the expression of antioxidant enzymes, thereby mitigating oxidative stress and neuroinflammation. The study demonstrates that the activation of the AMPK/Nrf2 and SIRT3/FoxO3a pathways leads to reduced α-synuclein oligomerization and necroptotic cell death in the substantia nigra area of a Parkinson’s disease mouse model, ultimately contributing to neurobehavioral recovery and neuroprotection against 1-methyl-4-phenyl-1,2,3,6-tetrahydropyridine-induced neurotoxicity (Leem et al., 2024).

### 6.3 Stroke and ischemic injury

Research has demonstrated that exercise can modulate various signaling pathways to promote neuroprotection and recovery following stroke and ischemic injury. This section highlights recent studies exploring the regulatory mechanisms involving FOXO proteins in these contexts (Table 3).

A study investigated the role of the AMPK/FOXO3a/SKP2/CARM1 signaling pathway activated by exercise pretreatment before ischemic stroke in mice. This pathway enhances transcriptional activity of transcription factor EB (TFEB), improving autophagic flux in the peri-infarct cortex. Exercise-induced AMPK activation increases FOXO3a phosphorylation, stabilizing CARM1, which binds to TFEB to enhance its transcriptional activity. This process reduces neuroinflammation, oxidative stress, and apoptosis, resulting in improved neurological function and reduced brain injury. Blocking TFEB or inhibiting AMPK nullified these protective effects, underscoring the significance of the AMPK-FOXO3a-TFEB pathway in exercise-mediated stroke protection (Zhao Y. et al., 2023).

Another study examined the PI3K/AKT/FoxO1 signaling pathway activated by exercise post-conditioning following ischemic stroke in rats. Exercise post-conditioning increased phosphorylation of PI3K, AKT, and FoxO1, promoting cytoplasmic retention of phosphorylated FoxO1 and thereby reducing its nuclear activity. This reduction in FoxO1 nuclear translocation suppressed the expression of gluconeogenic enzymes PCK-1 and PCK-2, lowering oxidative stress, apoptosis, and glucose levels. The findings indicate that exercise post-conditioning improves neurological function and reduces infarct volume by inhibiting gluconeogenesis through the PI3K/AKT/FoxO1 pathway (Li et al., 2023).

In conclusion, these studies highlight the critical role of FOXO signaling pathways in mediating exercise-induced neuroprotection and recovery following ischemic injury. Targeting these pathways through exercise-based interventions may offer promising therapeutic strategies for enhancing post-stroke recovery.

### 6.4 Miscellaneous: muscle-brain crosstalk and metabolic control

In the study by Liu et al., the FoxO protein is regulated through the FoxO signaling pathway during resistance exercise, specifically mediated by the protein kinase Sgk1. The RNA sequencing analysis revealed that Sgk1 is consistently upregulated in the frontal cortex, hippocampus, and muscle, with significant association to pathways related to learning, memory, and cognition. Further analysis indicated that Sgk1 is particularly enriched in the FoxO signaling pathway, which is recognized as the most significantly altered pathway among the tissues examined. This regulation promotes synaptic plasticity, improved cognitive performance, and enhanced mental health. Additionally, Sgk1 was identified as a key myokine likely contributing to the muscle-brain crosstalk mechanism. The positive correlation between Sgk1 expression and cognitive performance suggests its potential role as a candidate myokine for improving cognition and mental health through resistance exercise (Liu et al., 2024).

In Khoramipour’s study, the FoxO1 protein is regulated through the leptin signaling pathway after 8 weeks of HIIT in male rats with T2DM. HIIT enhanced hypothalamic leptin receptor expression and activated the downstream JAK2/STAT3 pathway, leading to increased phosphorylation of STAT3. Phosphorylated STAT3 promotes the expression of anorexigenic neuropeptides such as POMC and CART, while simultaneously inhibiting orexigenic neuropeptides. Additionally, HIIT reduced the expression of suppressor of cytokine signaling (SOCS3) and FoxO1 in the hypothalamus, thereby relieving the inhibition of anorexigenic pathways. Reduced FOXO1 activity, achieved through its phosphorylation and exclusion from the nucleus, allowed increased POMC gene expression, contributing to improved appetite regulation. The study found that HIIT improved hypothalamic appetite regulation by enhancing leptin signaling and downregulating FOXO1, which is crucial for appetite control in T2D rats (Khoramipour et al., 2023).

## 7 Exercise, metabolism, and FoxO-mediated systemic health

In this section we summarized the current data in Table 4 to illustrate that both aerobic and resistance-based protocols, alone or combined with nutritional or molecular interventions, can shift FoxO activity toward a more phosphorylated or otherwise inhibited state. This shift curbs gluconeogenic and lipogenic gene expression, enhances mitochondrial quality control, and ultimately improves glucose tolerance, lipid handling, and inflammatory balance across multiple tissues, underscoring central role of FoxO signaling in the systemic benefits of regular physical activity.

**TABLE 4:** Overview of key studies investigating how different exercise interventions modulate FoxO signaling pathways, with implications for metabolism, insulin sensitivity, and related health outcomes.

Exercise type	Samples	FoxO regulation by exercise	Main findings	Refs
Insulin sensitivity and resistance				
High-intensity interval training	19 control mice and 24 trained mice	↓ FoxO1 expression (inactivation)	HIIT modifies exosomal miRNA profiles, increasing miR-133b levels which suppress FoxO1 expression in the liver, improving insulin sensitivity and glucose tolerance.	Castaño et al. (2020)
Aerobic exercise	42 male mice	↑ FoxO1 phosphorylation (inactivation)	Exercise reduces FoxO1 and Pepck gene expression in prediabetic and diabetic mice, improving glucose metabolism and insulin sensitivity. In healthy mice, aerobic exercise increases Irs1 and Akt but does not affect FoxO1 or Pepck.	Kazeminasab et al. (2023)
Short-term strength training on a ladder apparatus	Male mice (8-weeks-old)	↑ FoxO1 phosphorylation (inactivation)	Short-term strength training increases hepatic FOXO1 phosphorylation, reduces PEPCK and G6Pase content, enhances hepatic insulin sensitivity, and improves glucose homeostasis in obese mice.	Pereira et al. (2022)
Obesity and Metabolic Dysfunction				
Acute exercise consisting of swimming.	9 male rats	↑ FoxO1 phosphorylation (inactivation)	Acute exercise increases FoxO1 phosphorylation, reducing PGC-1α interaction and PEPCK and G6Pase expression, improving insulin sensitivity in obese rats.	Ropelle et al. (2009)
Moderate incremental swimming exercise training	36 Senescence-accelerated mouse-prone 8 mice (6 months old)	↑ FoxO3 phosphorylation (inactivation)	Exercise and IF peptide enhance pAMPK/SIRT1/PGC-1α/pFOXO3 signaling, improving mitochondrial function, reducing apoptosis, and promoting cellular longevity in aging mice. This integrative approach provides better protection against HFD-induced cardiac and hepatic damages.	Asokan et al. (2020)
Intense exercise (GSE58559) Cycling (moderate-high intensity) (GSE116801) Moderate-intensity exercise (GSE43471)	105 samples were analyzed: GSE58559: 0 pre-exercise samples, 25 post-exercise samples. GSE116801: 10 pre-exercise samples, 10 post-exercise samples (male participants). GSE43471: 30 pre-exercise samples, 30 post-exercise samples (female participants)	↑ FoxO expression (activation)	Moderate exercise: Upregulates FOXO pathway, promotes adipose metabolism. Intense exercise: Increases inflammatory markers, disrupts immune balance. GF-1 involvement: Enhances FOXO1 protein in muscle/adipose tissue, promotes fat degradation	Lu et al. (2023)
Long-term resistance training by treadmill	60 postmenopausal women (55–70 years)	Did not affect FoxO1 regulation	Resistance training increased SIRT1 expression in PBMCs; DHA supplementation increased FOXO1. Exercise combined with DHA improved MASLD biomarkers and indices but had no significant effect on hepatic FOXO1 expression.	Yang et al. (2024)

↑ increased; ↓ decreased.

### 7.1 Insulin sensitivity and resistance

Physical exercise has been shown to modulate insulin sensitivity and resistance through various molecular pathways involving the FOXO1 protein. This section highlights recent studies investigating the regulatory mechanisms by which exercise impacts insulin signaling and glucose metabolism (Table 4).

In mice, a single mesocycle of HIIT provokes a surge in muscle-derived exosomes enriched for miR-133a and miR-133b. Once taken up by hepatocytes, these microRNAs directly suppress FoxO1 mRNA, thereby lowering PEPCK and G6Pase expression and normalizing glucose tolerance—even when canonical insulin signalling remains defective (Castaño et al., 2020). This exosomal mechanism is notable because it operates upstream of AKT and may therefore compensate for insulin-resistant states in which AKT activation is blunted.

By contrast, moderate-intensity continuous training relies on the canonical insulin–AKT pathway. Eight weeks of treadmill running in pre-diabetic or overtly diabetic mice markedly increases hepatic AKT phosphorylation, promotes FoxO1 phosphorylation at Ser256, drives its nuclear export, and diminishes transcription of PEPCK (Kazeminasab et al., 2023). Interestingly, the same protocol in healthy mice raises IRS-1 and AKT activity without further altering FoxO1 localization, suggesting that a metabolic “ceiling effect” limits additional repression once FoxO1 is already maximally inhibited. Thus, aerobic training appears to restore, rather than simply augment, AKT-FoxO1 coupling when insulin resistance is present.

Resistance exercise adds a third layer. Short-term ladder climbing performed by obese mice enhances both AKT and GSK3β phosphorylation in the liver, thereby phosphorylating FoxO1 and reducing PEPCK and G6Pase expression. However, this process occurs without altering adiposity or circulating insulin levels (Pereira et al., 2022). These data imply that mechanical–load–derived cues can directly reprogram hepatic glucose output, separating the glycemic benefits of resistance training from weight loss per se—a clinically relevant distinction for individuals who struggle to reduce fat mass.

Taken together, the three modalities reveal a pattern of mechanistic divergence that nonetheless converges on the inhibition of FoxO1. HIIT delivers endocrine microRNAs that silence FoxO1 transcripts; endurance exercise restores AKT-mediated phosphorylation of FoxO1 in insulin-resistant livers; resistance exercise mobilizes mechano-sensitive pathways that also activate AKT and collateral regulators, such as GSK3β. Each route may dominate or fail depending on the patient’s metabolic phenotype, disease duration, or even age- and sex-related differences in exosome biogenesis, or insulin signalling fidelity. Collectively, current evidence suggests that HIIT, continuous aerobic training, and resistance exercise all enhance insulin sensitivity by suppressing hepatic FoxO1.

### 7.2 Obesity and metabolic dysfunction

The regulation of FoxO proteins through various exercise regimes and nutritional interventions has been a topic of extensive research. This section summarizes key studies that investigate how FoxO proteins, particularly FoxO1 and FoxO3, respond to exercise, dietary supplements, and their combined effects on metabolic health (Table 4).

Rodent endurance work provides the evidence that acute aerobic bouts blunt FoxO-driven gluconeogenesis. In the classic swimming study by Ropelle et al., diet-induced obese rats underwent a single session of moderate-intensity swimming, resulting in the rapid re-phosphorylation of hepatic Akt. It forces FoxO1 into the cytoplasm and suppresses PEPCK/G6Pase transcription (Ropelle et al., 2009). The same stimulus downregulated PGC-1α, weakening its co-activator synergy with FoxO1. Mechanistically, this illustrates a tight Akt/FoxO/PGC-1α triad in which endurance exercise acutely shifts liver metabolism from glucose production to storage. Notably, the effect size (approximately a 50% rise in FoxO1 Ser^256^ phosphorylation) was achieved without changes in total FoxO1 protein, underscoring that post-translational modification, rather than abundance, is the primary control point in the obese liver.

By contrast, chronic swimming combined with a bioactive peptide in senescence-accelerated mice rewired FoxO3 rather than FoxO1 (Asokan et al., 2020). Twelve weeks of progressive aquatic training elevated pAMPK, SIRT1, and PGC-1α, culminating in FoxO3 phosphorylation and cytoplasmic retention. The switch from an Akt-to-AMPK-centered axis highlights that the upstream kinase controlling FoxO can diverge with training duration and energetic stress. Akt responds to insulin/glucose flux, whereas AMPK senses muscle ATP depletion. Notably, suppression of cardiac and hepatic apoptosis in this ageing model aligns with FoxO3’s canonical role in mitochondrial quality control, suggesting that endurance exercise curbs oxidative damage via AMPK/SIRT1/FoxO3 signalling rather than the Akt/FoxO1 route dominant in the obese but otherwise young rat.

Moving from muscle–liver cross-talk to adipose tissue, Lu et al. mined three transcriptomic datasets spanning intense cycling in humans and treadmill running in mice (Lu et al., 2023). Moderate-intensity bouts upregulated a FoxO-centered lipid-catabolic genes and improved adipose immune tone; in contrast, maximal efforts triggered a broad inflammatory transcriptome, including NF-κB and NLRP3 components, despite further FoxO activation. The observation that the FoxO pathway itself is not anti-inflammatory under high-load conditions emphasizes intensity-specific bifurcation. FoxO can sit upstream of both favorable (lipolytic) and adverse (inflammatory) outputs, depending on the surrounding signal milieu. The inter-species concordance—similar FoxO target enrichment in mouse and human adipose tissue, despite distinct exercise modes—supports translational relevance but also highlights the need for dose-finding studies in humans.

Human resistance data remain scarce. However, the 16-week treadmill-based strength protocol in postmenopausal women begins to close the gap (Yang et al., 2024). Training alone raised peripheral-blood-mononuclear-cell SIRT1 without altering FoxO1 mRNA, whereas DHA co-supplementation modestly increased FoxO1 expression but did not change hepatic FoxO1 activity. Clinically, the combined intervention improved MASLD biomarkers, indicating that metabolic benefit can accrue even when FoxO regulation is incomplete. The dissociation between circulating-cell expression and hepatic action echoes a recurrent translational barrier: tissue-specific FoxO status is rarely accessible *in vivo*, complicating the interpretation of human trials.

Across these studies, two mechanistic themes emerge. First, post-translational gating dominates FoxO control. Phosphorylation by Akt or AMPK (with SIRT1-mediated deacetylation as a parallel modifier) determines subcellular localization and transcriptional output, while changes in total FoxO protein are secondary. Second, exercise intensity and duration re-route the upstream kinase axes, shifting FoxO from an insulin-sensitive gluconeogenic repressor (Akt-FoxO1) toward an energy-sensing mitophagy regulator (AMPK-FoxO3). Diet augments this re-routing—either by providing antioxidant peptides that amplify AMPK signalling (Asokan et al., 2020) or by delivering n-3 PUFAs that selectively boost FoxO1 transcription (Yang et al., 2024). Notably, endurance paradigms in rodents converge on improvements in insulin sensitivity and organelle quality.

In contrast, early human studies suggest a broader metabolic benefit that is only partially dependent on FoxO. This divergence highlights a translational gap. Rodents permit tissue biopsies and kinase mapping, whereas human trials must rely on blood surrogates and imaging, thereby limiting mechanistic resolution.

Collectively, the data indicate that FoxO proteins function as context-dependent metabolic switches, whose activity is modulated by the type and dose of exercise, as well as concurrent nutritional cues. Endurance swimming and moderate cycling preferentially engage Akt- or AMPK-driven FoxO phosphorylation to suppress gluconeogenesis, stimulate lipolysis, and enhance mitochondrial defense, whereas resistance-oriented interventions in humans achieve metabolic benefits even with incomplete FoxO modulation. Going forward, integrating tissue-targeted biomarkers into human trials and systematically mapping kinase-specific FoxO signatures across various exercise intensities will be crucial to translating these mechanistic insights into precise, clinically actionable prescriptions for metabolic disease.

## 8 Discussion

Animal studies investigated the pharmacologically induced muscle atrophy. However, examining this theory in humans is difficult because these patients may not be able to exercise. Some studies have shown that exercise may inhibit muscle atrophy without affecting the FoxO signaling, e.g., in ICU patients.

The present review highlights the multifaceted impact of physical exercise on FoxO signaling pathways, emphasizing its role in cellular homeostasis, muscle adaptation, metabolic regulation, and neuroprotection. However, some limitations warrant consideration.

Firstly, a significant portion of the studies reviewed are based on animal models, which may not fully replicate the complexity of human physiology. The extrapolation of these findings to human subjects remains a challenge, particularly in understanding how exercise-induced modulation of FoxO pathways varies across species, age groups, sexes, and clinical conditions. Additionally, while preclinical studies provide valuable insights into the molecular mechanisms underlying FoxO signaling, the lack of longitudinal studies and randomized controlled trials in humans limits the ability to establish causal relationships between exercise interventions and clinical outcomes.

Secondly, the heterogeneity of exercise protocols used across studies complicates the interpretation of results. Factors such as exercise type (e.g., aerobic, resistance, high-intensity interval training), duration, frequency, and intensity are inconsistently reported, making it difficult to determine optimal exercise prescriptions for modulating FoxO pathways effectively.

Furthermore, the focus of most studies on skeletal muscle and cardiac muscle leaves gaps in understanding the systemic impact of exercise on other tissues where FoxO signaling plays a critical role, such as adipose tissue, liver, and the nervous system. This limits the development of comprehensive, targeted therapeutic strategies for conditions like metabolic syndrome, neurodegenerative diseases, and cardiovascular disorders.

Future research should aim to address these limitations by conducting well-designed clinical trials to validate findings from animal studies in human populations. Comparative studies that investigate how different exercise modalities influence FoxO signaling across various tissues are particularly needed. Moreover, exploring the interplay between FoxO signaling and other molecular pathways under diverse physiological and pathological conditions may provide deeper insights into the holistic benefits of exercise.

Finally, advancements in technologies such as single-cell RNA sequencing and proteomics could enable a more detailed understanding of the cell-specific regulatory mechanisms of FoxO proteins in response to exercise. This knowledge will be essential for developing precise, individualized exercise regimens aimed at optimizing health outcomes through modulation of FoxO pathways.

## 9 Conclusion

The present review underscores the critical role of FoxO signaling pathways in mediating the beneficial effects of physical exercise across various biological systems. Through its influence on cellular homeostasis, muscle adaptation, metabolic regulation, neuroprotection, and cardiovascular health, FoxO signaling emerges as a pivotal mechanism by which exercise exerts its multifaceted benefits. Exercise-induced modulation of FoxO pathways involves intricate interactions with upstream regulators such as PI3K/AKT, AMPK, and SIRT1, resulting in downstream effects that promote antioxidant defense, autophagy, mitochondrial function, and anabolic signaling.

Despite extensive research, much of the evidence is derived from animal models, necessitating further exploration through human-based studies to confirm and expand upon these findings. Additionally, the diversity of exercise protocols employed in studies highlights the need for standardized approaches to better understand the optimal conditions for FoxO modulation. Future research aimed at integrating findings from different tissues and disease models will be essential for developing targeted exercise interventions that harness FoxO signaling to improve health and combat disease.

Ultimately, enhancing our understanding of how exercise modulates FoxO pathways could unlock new avenues for therapeutic interventions aimed at improving metabolic health, mitigating muscle wasting, promoting neuroprotection, and enhancing cardiovascular resilience. Given the broad impact of FoxO signaling on human physiology, continued investigation into its regulation by exercise remains a promising frontier in the pursuit of health optimization and disease prevention.
