# Supplementary material for: The multifaceted impact of physical exercise on FoxO signaling pathways
Source: Front Cell Dev Biol. 2025 Aug 8;13:1614732. doi: 10.3389/fcell.2025.1614732 (PMC12370687; doi:10.3389/fcell.2025.1614732)
Supplement: Supplementary file 1 [file Table1.docx]

**Table S1**. Summary of studies examining the effects of various exercise protocols on FoxO signaling in skeletal muscle. Each entry details the exercise type, subject groups, regulatory changes in FoxO, and the key findings related to muscle adaptation and protein turnover.

| **Exercise Type** | **Samples** | **FoxO regulation by exercise** | **Main Findings** | **Refs** |
| --- | --- | --- | --- | --- |
| **Pharmacologically‐Induced Muscle Atrophy** | | | | |
| Aerobic training (treadmill) Resistance training (ladder climbing) | Male mice (8-week-old) | ↑ FoxO3a phosphorylation (inactivation) | Exercise reverses Dexamethasone-induced muscle atrophy by inhibiting HDAC4/FoxO3a signaling, increasing AKT phosphorylation, and reducing MuRF1 and Atrogin-1 expression​. Combined aerobic and resistance exercise improves muscle mass and function in Dexamethasone-treated mice, enhancing recovery​. Exercise increases FoxO3a phosphorylation, reducing its nuclear localization and transcriptional activity, thereby lowering muscle atrophy markers​. | (21) |
| Resistance exercise (incremental ladder climbing) | 40 male mice (8-week-old) | ↑ FoxO3a phosphorylation (inactivation) | Resistance exercise increased Sesn2 expression, inhibited FoxO3a nuclear translocation, and reduced muscle atrophy via decreased MuRF1 and Atrogin-1 expression. Sesn2 suppressed FoxO3a and MSTN/Smad pathways, enhancing muscle hypertrophy and protecting against dexamethasone-induced muscle loss. | (22) |
| Aerobic exercise (Treadmill) Resistance exercise (Ladder climbing) | 34 male rats (8-week-old) | ↑ FoxO3a phosphorylation (inactivation) | Both aerobic and resistance exercise reduced cisplatin-induced muscle atrophy by regulating the AKT/PGC1-α/FoxO3a pathway. Exercise increased phosphorylation of AKT, FOXO3a, and PGC1-α, decreasing autophagy markers (BNIP3, Beclin 1, p62, LC3-II/I ratio). Reduced muscle atrophy markers (MuRF1, Atrogin-1) through exercise in gastrocnemius and soleus muscles. Both exercise types were effective, but aerobic exercise was more dependent on FoxO3a-mediated degradation pathways. Exercise-induced PGC1-α upregulation plays a crucial role in preventing muscle atrophy. | (23) |
| Treadmill exercise | Male mice (8 weeks-old) | ↑ FoxO3a phosphorylation (inactivation) | Treadmill exercise restored Akt and FoxO3a phosphorylation inhibited by cisplatin. Exercise suppressed cisplatin-induced upregulation of MuRF1 and atrogin-1 in muscle tissue. Exercise attenuated the increase in myostatin gene expression induced by cisplatin. Cisplatin-induced phosphorylation of Smad2 was reduced by treadmill exercise. Exercise improved muscle mass and myofiber diameter reduced by cisplatin. | (24) |
| Endurance exercise | 36 male mice (8-week-old) | ↑ FoxO3a phosphorylation (inactivation) | Endurance exercise mitigated Doxorubicin-induced FoxO3α activation by restoring phosphorylation. Muscle-specific ubiquitin ligases (MuRF-1, MAFbx) and autophagy-related markers were not altered by Endurance exercise or Doxorubicin. Endurance exercise improved muscle quality and regeneration capacity, particularly through PAX7 activation and α-ACTN restoration​ | (25) |
| Short-term endurance exercise training (treadmill) | 24 male rats (6 months old) | ↑ FoxO3a and FoxO1 phosphorylation (inactivation) | Exercise attenuated Doxorubicin-induced FoxO1 and FoxO3 activation. Reduced MuRF-1 and BNIP3 expression in soleus muscle. Increased PGC-1α likely mediates protection by inhibiting FoxO transcription. Short-term exercise protects against Doxorubicin-induced muscle atrophy. | (26) |
| Aerobic training (treadmill) Resistance training (ladder climbing) | 40 male rats | ↑ FoxO3a phosphorylation ↓ Fox3a/O1 levels (inactivation) | Resistance training reduced FoxO1/3a expression. Resistance training significantly increased FoxO3a phosphorylation. Exercise suppressed FoxO-driven muscle atrophy genes (Atrogin-1, MuRF1, Myostatin). Resistance training most effectively mitigated muscle atrophy induced by Dapagliflozin treatment. | (27) |
| **Condition-Related Muscle Atrophy** | | | | |
| Moderate-intensity treadmill exercise | 36 mice (24 diabetic; 12 healthy) | **Did not affect FoxO3a regulation** | Moderate exercise suppressed NF-κB signaling and enhanced SIRT1-AMPK-PGC1α activity, promoting mitochondrial biogenesis and reducing MuRF-1 expression. The intervention improved muscle health by inhibiting inflammation-induced atrophy but did not directly affect FoxO3a phosphorylation. | (28) |
| Resistance training (Muscle Overloading),  Endurance training (Treadmill) | Male mice with CKD | ↑ FoxO1 phosphorylation (inactivation) | Muscle overloading significantly improved muscle protein synthesis and phosphorylation of FoxO1, while treadmill running suppressed protein degradation. Both training types reduced CKD-induced FoxO1 activation, but overloading was more effective in promoting muscle hypertrophy and enhancing protein synthesis. | (29) |
| Swim training | Transgenic male mice | ↓ Fox3a expression (inactivation) | Swim training decreased FoxO3a activation, reduced MuRF1 levels, and partially reversed muscle atrophy. No significant changes were noted in mTOR/p70S6K pathway proteins. | (30) |
| Resistance exercise (ladder climbing) | 40 male rats (8-week-old) | ↓ FoxO1 expression ↑ FoxO1 cytoplasmic deacetylation  (inactivation) | Resistance training decreases nuclear FoxO1, cytoplasmic acetylated FoxO1, and inhibits autophagy-induced muscle atrophy​ | (31) |
| Mice: Forced wheel running Worms: Swimming in M9 buffer | Mice: Male (16 months old) Worms (C. elegans): N2 Bristol strain | ↑ FoxO3a transcriptional activity (activation) | Exercise activates AdipoR1–AMPK–FoxO3a pathway enhancing autophagy, mitophagy, muscle mass, and quality; prevents senescence; extends lifespan in worms. | (32) |
| Functional electrical muscle stimulation | **Critically ill patients on mechanical ventilation in ICU Metabolically healthy volunteers** | **Did not affect FoxO regulation** | Functional electrical muscle stimulation did not improve muscle function or reduce muscle wasting in critically ill patients. Altered FoxO-mediated transcription was observed but was not improved by Functional electrical muscle stimulation. Persistent intramuscular inflammation and altered substrate utilization were noted. No significant differences in outcomes between Functional electrical muscle stimulation and standard care groups | (33) |
| **Diet and Lifestyle Factors** | | | | |
| Resistance training (climbing) | Drosophila of different genetic backgrounds | ↑ FoxO expression (activation) | Exercise enhances FoxO/PGC-1α/SDH and FoxO/SOD pathways, improving mitochondrial function and reducing oxidative stress​. FOXO overexpression enhances resistance against high-salt-induced aging, while FoxO-RNAi blocks exercise benefits​. FoxO mediates exercise-induced protection against age-related decline in cardiac and muscle function | (34) |
| Endurance exercise (treadmill) | Female rats (4–6 weeks old) | ↑ FoxO3a and FoxO1a phosphorylation (inactivation) | Extra Virgin Olive Oil + training reduced phosphorylated FoxO3a, enhancing autophagy and improving muscle integrity. High-fat diet increased phosphorylation of FoxO3a, reducing its nuclear activity and contributing to muscle degradation. Exercise training activates FoxO signaling and improves mitochondrial homeostasis. | (35) |
| Resistance exercise | Age-matched male Drosophila flies | ↑ FoxO expression (activation) | Exercise enhances FoxO activity via Sirt1/FoxO/SOD/CAT pathways​. Resistance training reduces lipid accumulation and improves antioxidant capacity​. FoxO is not a key regulatory gene for resistant training against damage, but plays a role in high-fat diet-induced damage | (36) |
| Aerobic exercise (swimming) | 100 healthy male zebrafish (7-month-old) | ↑ FoxO3a phosphorylation (inactivation) | Aerobic exercise down-regulates miR-128, activates IGF-1/PI3K/AKT signaling, phosphorylates FoxO3a, enhances mitochondrial function, improves sarcopenia​. | (37) |
| Treadmill running, Resistance exercise (ladder climbing), Alternating treadmill and resistance exercise, Voluntary wheel running​ | 60 male rats (21 months) | **↓ FoxO3a phosphorylation (activation)** | Exercise modulates Akt/FoxO3a signaling pathway, enhancing autophagy and reducing apoptosis in aged rats​. | (38) |
| **Transcriptomic and Gene Expression Adaptations** | | | | |
| Moderate-intensity continuous training and High-intensity interval training | Male mice (14-week-old) | ↑ FoxO1 phosphorylation (inactivation) | Moderate-intensity training increases IGF-1 expression, enhancing PI3K-AKT-FoxO signaling, which downregulates FoxO1 activity and potentially reduces muscle atrophy. High-intensity interval training shows higher FoxO1 expression linked to oxidative stress and muscle catabolism, suggesting different molecular adaptations between these training modalities. | (39) |
| Resistance exercise | **12 participants (10 males and 2 females) (19 to 45 years)** | ↓ FoxO3a expression (inactivation) | Exercise reduces FoxO3a mRNA levels, especially at 4h post-exercise. Heat application does not significantly influence FoxO3a expression | (40) |
| Eccentric and Concentric resistance training | **24 healthy, untrained young men (23.9 years)** | ↑ FoxO3a and FoxO1 phosphorylation (inactivation) | Eccentric training increased FoxO1 and FoxO3a phosphorylation, reducing nuclear translocation and transcription of atrophy genes. This suggests Eccentric training is effective for muscle growth and protection against degradation | (41) |
| Acute resistance exercise (lumbar extension exercise) | **64 participants undergoing spinal surgery** | ↓ FoxO3a expression (inactivation) | Acute resistance exercise reduced FoxO3 expression but did not enhance myogenesis in lumbar spine pathology patients. Instead, inflammation and extracellular matrix adaptation pathways were activated, suggesting impaired muscle adaptability. This response highlights challenges in utilizing exercise-based interventions for muscle recovery in individuals with lumbar spine pathology. | (42) |
| High-intensity interval training and subsequent detraining (7-day and 14-day) | 24 male rats | ↓ FoxO3a expression (inactivation) | High-intensity interval training reduced FoxO3a mRNA but increased NF-κB and MuRF1. FoxO3a mRNA increased during detraining, promoting muscle atrophy. PGC1α expression increased after HIIT but decreased during detraining. The FoxO3a/MuRF1 pathway is more influential in atrophy than NF-κB/MuRF1. | (43) |
| **Mitochondrial Function and Autophagy** | | | | |
| Incremental load swimming training to exhaustion | 30 Male rats (6–8 weeks old) | ↑ FoxO3 expression (activation) | Cannabidiol inhibits excessive autophagy/mitophagy via FoxO3/GABARAPL1, PINK1/PARKIN, and BNIP3 pathways, improving mitochondrial function in exhaustive exercise-induced skeletal muscle damage. | (44) |
| Hypoxic training combined with treadmill exercise under normoxia | Male mice aged (8-weeks-old) | ↑ FoxO3 expression (activation) | Hypoxic training upregulates Sirt3, enhancing FoxO3a activation which promotes mitophagy and antioxidant responses. Sirt3 inhibition reduces FoxO3a levels and impairs mitophagy signaling. FoxO3a regulation via hypoxic training is partially maintained even when Sirt3 activity is inhibited. | (45) |
| Aerobic exercise, resistance exercise (unilateral leg extensions)​ | **Six healthy, untrained men (27 ± 3 years)​** | ↑ FoxO3a activation (activation) | Acute RE more effectively activates FoxO3 at 4 hours post-exercise compared to AE. Both AE and RE induce early FoxO3 activation at 1-hour post-exercise. Greater expression of autophagy-related genes following RE indicates a more substantial FoxO3-driven response | (46) |
| Acute endurance exercise (cycling). | **Total participants: 27 middle-aged men/ diabetic group: 13 overweight/obese male patients with diabetes,  Control group: 14 weight-matched, healthy, non-diabetic male participants.** | ↑ FoxO3a phosphorylation (inactivation) | Exercise increased FoxO3a phosphorylation in diabetic patients and controls. Enhanced autophagy signaling through ULK1 phosphorylation. Reduced LC3B-II indicates increased autophagic turnover. Autophagy responses are intact in diabetic patients. | (47) |
| **Signaling Pathways and Molecular Markers** | | | | |
| Aerobic training on a treadmill, at moderate-to-high intensity | 36 female rats (8 months old) | ↑ FoxO1 expression (inactivation) | Lifelong aerobic training suppresses FoxO1 and MAFbX expression, enhancing muscle mass by promoting protein synthesis via PI3K/AKT/mTOR pathway. Detraining upregulates FoxO1, increasing protein degradation. Lifelong aerobic training’s inhibition of the FoxO pathway is reversed by detraining, promoting muscle atrophy | (48) |
| Acute and chronic eccentric exercise (downhill running) | 24 young male rats | Acute Eccentric Exercise: ↑ FoxO1 expression (activation) Chronic Eccentric Exercise: ↓ FoxO1 expression (inactivation) | Acute eccentric exercise increased FoxO1 mRNA expression in fast-twitch vastus lateralis muscle. Chronic eccentric exercise decreased FoxO1 expression due to adaptation. FoxO1 upregulation correlates with fast-twitch muscle adaptation. Muscle fiber type transition is influenced by oxidative stress. | (49) |
| Resistance exercise | **19 physically active, non-sarcopenic, older adults (65–78 years; 7 females and 12 males).** | ↑ FoxO3a phosphorylation ↓ FoxO1 expression (inactivation) | Resistance exercise increased CB2 expression linked to improved muscle maintenance via FoxO3a activation and FoxO1 suppression. Increased CB2 expression was positively associated with myogenic markers MyoD and Pax7, suggesting improved muscle regenerative capacity in older adults. | (50) |
| Mild endurance treadmill exercise | 16 male rats (12 weeks old) | ↑ FoxO1 phosphorylation (inactivation) | Mild endurance exercise during fasting increased FoxO1 phosphorylation, enhancing muscle recovery via BDNF-CREB and Akt-mTOR signaling. Increased deiodinase 2 expression and decreased deiodinase 3 expression raised T3 levels, promoting muscle quality. Exercise partially restored mTORC1 signaling suppressed by fasting. | (51) |
| **Muscle Remodeling, Growth, and Adaptation** | | | | |
| Resistance exercise performed on a single leg | **Healthy, untrained early postmenopausal women (within five years post-menopause)** | ↑ FoxO3a and FoxO1 phosphorylation (inactivation) | Red clover extract supplementation reduced FoxO1 and FoxO3a levels, reducing muscle protein degradation​. Higher p-Akt levels post-exercise suggest inhibition of FoxO-induced proteolysis. Red clover extract increases HSP27 expression, which may protect muscle fibers against degradation. Atrogin-1 protein content was significantly lower following Red clover extract, indicating reduced protein degradation. | (52) |
| Cold Water Immersion after resistance exercise on one leg | **9 male participants** | ↓ FoxO3a expression (inactivation) | Post-exercise cooling and active recovery decrease FoxO3a levels in muscle without significantly affecting phosphorylation or nuclear localization. | (53) |
| Moderate-intensity continuous training (treadmill) | 8-month-old and 26-month-old rats | ↑ FoxO1 phosphorylation (inactivation) | Lifelong moderate-intensity training upregulates phosphorylated FoxO1, enhancing SOD2 activity, reducing oxidative stress, and promoting muscle regeneration. | (54) |
| Cold water immersion after whole-body resistance training | **16 recreationally active men (22.9 ± 4.6 years)** | **↓ FoxO1 phosphorylation (activation)** | Cold water immersion increased basal FoxO1 protein content, promoting protein degradation. FoxO3a phosphorylation decreased post-exercise but was unaffected by cold water immersion. Muscle hypertrophy was compromised by cold water immersion, while strength gain was not. Increased protein degradation was suggested as a key mechanism. | (55) |
| Acute treadmill exercise (aerobic) | 5-6 mice per group (REDD1 KO and WT) (3–4 months) | **Did not affect FoxO1/O3a regulation** | Acute exercise reduced mTORC1 signaling via AMPK activation. REDD1 KO mice showed improved insulin-stimulated signaling but impaired FoxO phosphorylation. mTORC1 hyperactivation persisted despite exercise, partially limiting insulin signaling improvements. REDD1 deficiency compromised FoxO activation response to exercise. | (56) |
| Aerobic exercise (treadmill) | **17 free-living, recreationally active males** | ↓ FoxO3a expression (inactivation) | Carbohydrate intake increased phosphorylated AKT and phosphorylated FoxO1 levels, reducing proteolysis. Lower PTEN and MURF1 expression with carbohydrate intake. Enhanced miRNA targeting PI3K-AKT, FoxO, mTOR pathways. Reduced muscle protein breakdown with carbohydrate ingestion. | (57) |
| High-intensity running | 32 male mice (5-week-old) | **↓ FoxO1 and FoxO3a phosphorylation  (activation)** | Running inhibits AKT activation, leading to dephosphorylation of FoxO1 and FoxO3a, promoting autophagy in mdx mice | (58) |
| Treadmill running (resistance training) | 35 albino rats (8 weeks old) | ↓ FoxO3a and FoxO1 expression (inactivation) | SAC supplementation reduced FoxO1/FoxO3 and ubiquitin–proteasome pathway activity, enhancing muscle protein synthesis via increased IGF-1, Akt, mTOR, 4E-BP1, and S6K1. The combined supplement group (E + S + A + Cr) showed the most pronounced effects. | (59) |
| Resistance training (bodyweight and elastic bands)​ | **38 sedentary, non-smoking participants (18 males and 20 females) (63.5 years)** | ↓ FoxO3a expression (inactivation) | Resistance training decreased FoxO3 activity via activation of Akt and related pathways, enhancing muscle function and composition. This adaptation likely promotes muscle maintenance through a shift from oxidative type I to fast type IIA muscle fibers. | (60) |
| Progressive aerobic exercise (cycling) | **9 old women (70 years)** | ↓ FoxO3a expression (inactivation) | Aerobic training reduced FOXO3A mRNA by 24% without affecting protein levels. Increased mitochondrial protein (COX IV) by 33%. Reduced PGC-1α protein content by 20%. Improved muscle health potentially linked to reduced proteolytic signaling. | (61) |
| **Muscle-Specific Studies: Recovery and Hypertrophy** | | | | |
| High-intensity interval training on a treadmill with upslope and downslope running | 30 male rats (20 weeks old) | ↓ FoxO3 expression (inactivation) | High-intensity interval training combined with BCAA/nano chitosan reduced FoxO3 and SMAD expression, particularly in the Upslope + Sup group. Supplementation enhanced muscle recovery and structure by inhibiting muscle degradation pathways, indicating a potential strategy to counteract aging-related muscle atrophy. | (62) |
| High-intensity interval training | 12 male rats (10 weeks old) | ↓ FoxO1 expression (inactivation) | High-intensity interval training reduced FoxO1 expression and increased IGF-I/Akt signaling, promoting muscle hypertrophy. Downregulation of myostatin/Smad pathways contributed to muscle growth, with significant increases in muscle cross-sectional area and weight. | (63) |
| Resistance training | 96 male rats (6-weeks-old) | ↓ FoxO3 expression (inactivation) | TGF‐β1 inhibition enhances muscle hypertrophy via modulation of Smad signaling, indirectly affecting FoxO proteins​. | (64) |
| Progressive resistance training (ladder climbing) | **36 male rats** | ↓ FoxO3 phosphorylation (activation) | Resistance exercise restored soleus muscle degradation induced by myocardial infarction. Resistance exercise mitigated muscle damage, reduced inflammation, and promoted better muscle function and structure. | (65) |

↑ increased; ↓ decreased
